# A multidisciplinary approach to the antioxidant and hepatoprotective activities of *Arbutus pavarii* Pampan fruit; *in vitro* and *in Vivo* biological evaluations, and *in silico* investigations

**DOI:** 10.1080/14756366.2023.2293639

**Published:** 2023-12-28

**Authors:** Fatma A. Elshibani, Abdullah D. Alamami, Hamdoon A. Mohammed, Rabab Ahmed Rasheed, Radwa M. El Sabban, Mohamed A. Yehia, Sherif S. Abdel Mageed, Taghreed A. Majrashi, Eslam B. Elkaeed, Mahmoud A. El Hassab, Wagdy M. Eldehna, Mohamed K. El-Ashrey

**Affiliations:** a Department of Pharmacognosy, Faculty of Pharmacy, University of Benghazi, Benghazi, Libya; b Department of Basic Medical Science, Faculty of Pharmacy, University of Benghazi, Benghazi, Libya; c Department of Medicinal Chemistry and Pharmacognosy, College of Pharmacy, Qassim University, Qassim, Saudi Arabia; d Department of Pharmacognosy and Medicinal Plants, Faculty of Pharmacy, Al-Azhar University, Cairo, Egypt; e Department of Medical Histology and Cell Biology, Faculty of Medicine, King Salman International University (KSIU), South Sinai, Egypt; f Department of Anatomy, Faculty of Medicine, October 6 University, Giza, Egypt; g Department of Forensic Medicine and Clinical Toxicology, Faculty of Medicine, October 6 University, Giza, Egypt; h Pharmacology and Toxicology Department, Faculty of Pharmacy, Badr University in Cairo (BUC), Badr City, Cairo, Egypt; i Department of Pharmacognosy, College of Pharmacy, King Khalid University, Asir, Saudi Arabia; j Department of Pharmaceutical Sciences, College of Pharmacy, AlMaarefa University, Riyadh, Saudi Arabia; k Department of Medicinal Chemistry, Faculty of Pharmacy, King Salman International University (KSIU), South Sinai, Egypt; l Department of Pharmaceutical Chemistry, Faculty of Pharmacy, Kafrelsheikh University, Kafrelsheikh, Egypt; m Pharmaceutical Chemistry Department, Faculty of Pharmacy, Cairo University, Cairo, Egypt

**Keywords:** Oxidative stress, immunohistochemistry, libyan strawberry, phytochemicals, molecular docking

## Abstract

The Libyan Strawberry, *Arbutus pavarii* Pampan (ARB), is an endemic Jebel Akhdar plant used for traditional medicine. This study presents the antioxidant and hepatoprotective properties of ARB fruit-extract. ARB phytochemical analysis indicated the presence of 354.54 GAE and 36.2 RE of the phenolics and flavonoids. LC-MS analysis identified 35 compounds belonging to phenolic acids, procyanidins, and flavonoid glycosides. Gallic acid, procyanidin dimer B3, β-type procyanidin trimer C, and quercetin-3-O-glucoside were the major constituents of the plant extract. ARB administration to paracetamol (PAR)-intoxicated rats reduced serum ALT, AST, bilirubin, hepatic tissue MDA and proinflammatory markers; TNF-α and IL-6 with an increase in tissue GSH level and SOD activity. Histological and immunohistochemical studies revealed that ARB restored the liver histology and significantly reduced the tissue expression of caspase 3, IL-1B, and NF-KB in PAR-induced liver damage. Docking analysis disclosed good binding affinities of some compounds with XO, COX-1, 5-LOX, and PI3K.

## Introduction

Medicinal plants are a great global source of medications and have participated in the modern conventional medicinal system through direct application, use of their natural constituents in the treatment of various disorders, or use of these constituents as candidates for the new synthetic drug templates.^1^^,^^2^ The consequence of the contributions of natural product research to the modern medical system is that approximately 25% of the currently available drugs are natural-based or contain natural ingredients as part of the final medicinal product.^3^^,^^4^ There are regular demands for natural product-based drugs and natural supplements as alternatives to non-selective, more toxic, and less sensitive synthetic medicines.^5^

In the human body, there is a regular and required level for reactive oxygen species (ROS), which are produced as a normal byproduct of metabolic processes in cells and play several physiological roles, including their involvement in immune system activation and cell signalling.^6^ However, a high level of ROS in the body, which is produced owing to several internal (e.g., mitochondrial dysfunction) and external factors (e.g., pollution, radiation, certain foods, and drugs) over the body’s capacity to neutralise, is a crucial factor in the initiation of several inflammatory diseases affecting soft tissues of the body^7^^,^^8^ such as neurodegeneration, Alzheimer’s disease, liver dysfunction, infertility, ischaemic heart disease, diabetes, and kidney disease.^9–12^

The liver is one of those important soft tissues owing to its multiple crucial metabolic and detoxification functions in the body. It is also a factory, supplying the body with immunity, bile, and blood clotting factors.^13^ The liver is one of the most sensitive organs in the body for internal and external oxidative stress. In addition, ROS are produced at limited levels by the mitochondria and endoplasmic reticulum of the liver cells to induce their normal physiological functions.^14^ The overproduction of ROS in hepatocytes is directly controlled by specific antioxidant enzymes, e.g., superoxide dismutase (SOD), catalase (CAT), and glutathione peroxidase (GPx), as well as several other antioxidant compounds such as glutathione (GSH) and tocopherol as non-enzymatic ROS neutralising agents.^15^

The reduction in internal protective enzymatic and non-enzymatic antioxidant levels results in serious redox states and oxidation of cellular biomolecules, leading to acute and chronic liver disorders.^15^^,^^16^ Therefore, external antioxidants supplemented in the diet or nutritional products are very important, especially for those with oxidative-related degenerative disorders or who live in areas where external oxidative stress factors are dominant.^17^^,^^18^ In that context, most of the liver supports available on the market are antioxidant plant-based natural products.^19^ In addition, several plants, including *Silybum marianum*,^20^* Suaeda vermiculata*, and *Alhagi maurorum*^21^, are among the natural hepatoprotective herbs used by natural healers in the traditional medicine-based curing system.

Phenolic acids and flavonoids form the powerful antioxidant machinery of medicinal plants. They are widely distributed throughout the plant kingdom, but their abundance in a single plant indicates the plant’s potential antioxidant and other activities. They are also biosynthesized in greater quantities by plants growing in specific environmental conditions in order to play antioxidant defensive mechanisms within these plants.

The Libyan strawberry, *A. pavarii* Pampan (ARB), is an evergreen shrub endemic to the Libyan Green Mountain in Gebal Al-Akhdar. The fruit of ARB is used in honey production and food supplements and possesses potent antioxidant activity. Moreover, the ripe fruit contains appreciably high amounts of the two fat-soluble antioxidant vitamins A and E, exceeding the amount in the unripening stage, while a high concentration of vitamin C characterises the unripe one. Furthermore, several phenolic acids and flavonoids have been identified from the plant, such as arbutin, gallic acid, apigenin, epicatechin, hesperidin, kaempferol, naringin, quercetin, and rutin.^22^

We are providing in the present work a phytochemical identification for the phenolic and flavonoids of ARB fruits besides an *in vitro* evaluation of its antioxidant activity. The hepatoprotective effect of the fruit extract was also evaluated against paracetamol (PAR)-induced toxicity in rats in comparison to the commercially used N-Acetylcysteine (NAC). The study also includes an evaluation of the antioxidant and anti-inflammatory biomarkers as part of the underlying mechanism for the plant hepatoprotective activity.

Eventually, to study complex biological and chemical systems, pharmaceutical research has successfully incorporated a wealth of molecular modelling methods into a variety of drug discovery programs. The combination of computational and experimental strategies has proven extremely useful in discovering and developing novel promising compounds. Molecular docking methods, widely used in modern drug design, investigate the ligand conformations adopted within the binding sites of macromolecular targets.^23^

## Material and methods

### Plant materials

The fruit section of *A. pavarii* Pampan was collected from Al-Jabal Al Akhdar, El-Bieda city, Libya, in January 2021 and identified by the taxonomist in the Department of Botany, Faculty of Sciences, Benghazi University, Libya. The fruits were dried in the shade for three weeks and ground to a fine powder. The fruit powder is stored in tightly closed, amber-colored containers at refrigerator temperature.

### Extraction procedure

For the determination of phenolics and flavonoid contents as well as the biological assays: The dried and grinded fruits of the plant (285 g) were macerated overnight at room temperature (RT) with 0.75 L of the aqueous methanol mixture (80:20 v/v). The plant solvent mixture was vibrated at 150 rpm to facilitate phenolic and flavonoid extraction. The extract was filtrated, and the plant residue was re-extracted with the same solvent under similar conditions. The combined extracts were evaporated at 40 °C under reduced pressure to dryness. For the HPLC analysis, 1 g of the dried and grinded fruits was extracted by 30 ml of the aqueous methanol mixture previously described. The combined extract was only subjected to normal evaporation at 35 °C to remove methanol. The residual aqueous-based extract was semi-purified from highly polar substances and sugars as part of its preparation for the HPLC analysis by passing the extract through a C-18 SepPak® Vac 3 cc cartridge (Phenomenex) (solid phase extraction), previously activated with methanol followed by water. A 0.22-m disposable LC filter disc was used to filter the methanolic extract after it had been re-dissolved in 1 ml of 20% aqueous methanol and concentrated under vacuum.

### Total phenolic content

Folin-Ciocalteu’s technique was used to quantify the amount of total phenolic compounds present in the methanolic extract.^24^ At a wavelength of 760 nm, the blue colour’s intensity was assessed in comparison to distilled water, a blank.^25^ The procedure was completed in triplicate, and the results were represented as mg of gallic acid equivalents (mg GAE/g) per gram of dried fruit extract.

### Total flavonoid content

The method was conducted exactly according to the reported assay of the aluminium chloride (AlCl3)-flavonoids complex, which is expressed as a yellow colour and can be measured by the intensity of the colour at 420 nm.^26^ A blank experiment composed of methanol instead of plant extract was also prepared. The results, which were represented as mg rutin equivalents per gram dry weight (mg Rut/g), were made in triplicate.

### HPLC-DAD-ESI/MS analysis of the phenolics and flavonoids

A Hewlett-Packard 1100 chromatograph (Agilent Technologies) equipped with a quaternary pump and a diode array detector (DAD) was used to evaluate the semi-purified plant extracts. An HP Chem Station (ver. A.05.04) data processing station was connected to the machine. A Waters Spherisorb S3 ODS-2 C18, 3 µm (4.6 mm × 150 mm) column thermostated at 35 °C was used. 0.1% formic acid (A) and acetonitrile (B) were the two solvents employed in the gradient elution protocol of the sample components as follows: 5 min (10% B to 15% B), 5 min (15–25% B), 10 min (25–35% B), then the last 10 min was followed by the isocratic system of 50% B. The column flow rate was 0.5 ml/min. A mass spectrometer (MS) linked to an HPLC system through the DAD cell output and the DAD were used for double online detection with the recommended wavelengths of 280 nm and 370 nm. Authentic samples of phenolic acids and flavonoids were used in the identification of the extract constituents through a comparison of their retention times (RT); these constituents were assigned by ASTERISK (*) in Table 4.

**Table 4.** LC-MS-MS analysis of *Arbutus pavarii* Pampan fruits extract.

No	RT	Compounds	MS (m/z): [M-H]-	**%** ^b^	MS/MS, m/z
1	3.94	Gallic acid glucoside	331.0640	0.063	169, 211, 271^26^
2	9.66	Gallic acid	169.0137	0.784	125^27^
3	10.03	Galloyl quinic acid	343.0652	0.046	191^28^
4	10.15	Protocatecuic acid	153.0194	0.009	109^27^
5	10.60	Protocatechuic acid *O*-hexoside	315.0698	0.046	108, 153^26^
6	10.68	Vanillic acid-*O*-glucoside ester	329.0855	0.031	108, 123, 167, 219^26^
7	10.83	Galloyl shikimic acid^a^	325.0561	0.118	125, 169, 170^29^
8	10.90	Epigallocatechin	305.0649	0.244	125, 137, 165^27^
9	11.28	2,4-Dihydroxybenzoic acid	153.0198	0.118	109^30^
10	11.66	Procyanidin dimer B3^a^	577.1272	3.215	125, 161, 289, 407, 425, 577^31^
11	11.66	β-Type procyanidin trimer C^a^	865.1884	0.991	287, 575, 577, 695^31^
12	11.74	Vanillin	151.0403	0.006	123, 137^30^
13	12.08	Trigalloyl glucoside	635.0840	0.052	169^26^
14	12.12	Cyanidin-3-*O*-glucoside	449.1037	0.571	259, 287^26^
15	12.23	Methyl gallate	183.0285	0.679	78, 124, 168^32^
16	12.30	Digalloyl shikmic acid^a^	477.1514	0.002	169, 297, 196
17	12.38	3-Hydroxy benzoic acid	137.0236	0.003	93, 108^27^
18	12.38	Myricetin glucoside	479.0793	0.380	316
19	12.49	Quercetin 3-*O*- rutinoside (Rutin)^a^	609.1396	0.239	151, 300
20	12.61	Quercetin-*O*-galloyl-glucoside	615.0919	0.507	169, 300, 302, 463^26^
21	12.76	Procyanidin A1	575.1158	0.014	259, 452, 509
22	12.80	Luteolin-7-glucoside	447.0904	0.024	152, 285^33^
23	12.83	Quercetin-3-*O*-glucoside (Isoquercitin)^a^	463.0833	1.207	151, 179, 301^33^
24	13.06	Ellagic acid	300.9983	0.301	145, 201, 229, 299^32^
25	13.21	Quercetin-3-*O*-(arabinoside/xyloside)	433.0743	0.182	271, 300^26^
26	13.29	Isorhamnetin 3-*O*-glucoside	477.1003	0.063	243, 271, 315, 399^26^
27	13.33	Quercetin 3-*O*-rhamnoside (Quercitrin)^a^	447.0884	0.628	151, 255, 302
28	13.55	Myricetin rhamnoside^a^	463.0821	0.027	179, 301, 316, 317, 463^34^
29	13.82	Vitexin	431.0963	0.035	171, 285, 401, 431
30	14.08	Myricetin	317.0288	0.177	151, 178, 271^26^
31	14.12	Catechin^a^	289.0817	0.001	125, 178, 259, 287
32	14.80	Chrysin	253.0492	0.001	152^32^
33	15.03	Quercetin	301.0344	0.219	151, 179^30^
34	15.22	Luteolin	285.0420	0.278	133, 149, 175, 285^35^
35	15.98	Kaempferol	285.0396	0.137	211, 239, 285^30^
Total relative percentages				11.397	

^a^ Compounds identified by matching with retention time (RT) of standard authentic;

^b^ Relative abundance of the compounds in the plant extract based on peak areas of the identified compounds compared to the total peaks’ areas in the LC-chromatogram.

An ESI source, a triple quadrupole-ion trap mass analyser, and an API 3200 Qtrap (Applied Biosystems, Darmstadt, Germany) were used for the MS observation process. Analyst 5.1 was used to operate the apparatus according to the parameters mentioned in the literature.^36^

### .*Antioxidant assays*

#### DPPH (2,2‐diphenyl‐1‐picrylhydrazyl) scavenging activity (DPPH-SA)

The DPPH assay was used, as mentioned in the literature,^37^with slight modifications to suit the 96-well plates. Briefly, 150 µL of freshly prepared DPPH reagent (prepared by dissolving 2 mg with 51 ml of methanol HPLC grade) was mixed with plant extract (5 µL), and then the plate containing the mixture was incubated in the dark for 30 min at RT. At 517 nm, the change in DPPH colour was assessed in three independent measurements, and the DPPH-SA's equivalent to Trolox was determined.

#### Ferric reducing antioxidant power (FRAP) assay

The assay was carried out according to the reported method.^38^ In a 96-well plate, the TPTZ working reagent (190 μl) composed of acetate buffer (300 mM PH = 3.6), TPTZ (10 mM in 40 mM HCl), and FeCl3 (20 mM) was added to 10 μl of the plant extract, and the mixture was kept at RT for 30 min before being measured at 593 nm. The FRAP extract activity was measured as mg Trolox equivalent.

#### Oxygen radical absorbance capacity (ORAC) assay

The assay was carried out according to the reported method.^39^ 10 µL of the extract were incubated with 30 µL fluoresceine (100 nM) for 10 min at 37 C. For background measurement, three cycles of fluorescence measurement (485 EX, 520 EM, nm) were performed with a cycle length of 90 s. Then, each well received 70 µL of freshly prepared 2,2′-Azobis(2-amidinopropane) dihydrochloride (AAPH) (300 mM). The measurement of fluorescence (485 EX and 520 EM nm) was kept up for 60 min (40 cycles, each lasting 90 s).

### In Vivo study

#### Animal housing and ethical declaration

Thirty adult male albino rats (180:220 gm, 10:12 weeks) were purchased from National Cancer Institute (Cairo, Egypt), housed in stainless steel cages in a special room with sanitary conditions, at a constant temperature range (22–24 °C), with direct daylight and natural ventilation. Animals were allowed to acclimate for one week before the study with unlimited access to rat chow and tap water during the acclimation and experimental period. This study was conducted per NIH Guidelines for the Care and Use of Laboratory Animals and approved by the Research Ethics Committee of the Faculty of Pharmacy, Badr University in Cairo (BUC-REC), Egypt (Approval no. PO-111-A).

#### Medications

Paracetamol (PAR) and N-Acetylcysteine (NAC) in powder form and Tween 80 in liquid form were supplied from Sigma-Aldrich (St. Louis, MO, USA). All other chemicals and reagents used in the study were of analytical grade.

#### Experimental design

Thirty rats were randomly divided into five groups (*n* = 6), as follows: Control group: received 10% Tween 80 (10 ml/kg/day) for 28 days. ARB group: received a methanolic extract of ARB (500 mg/kg/day) for 28 days.^40^ PAR group: received 10% Tween 80 (10 ml/kg/day) for 28 days, followed by PAR (2 g/kg) suspended in 10% Tween 80.^41^^,^^42^ NAC + PAR group: received 10% Tween 80 (10 ml/kg/day) for 14 days, followed by NAC (100 mg/kg/day) dissolved in 10% Tween 80 for additional 14 days, followed by PAR (2 g/kg) suspended in 10% Tween 80^41^. ARB + PAR group: received methanolic extract of ARB (500 mg/kg/day) for 28 days followed by PAR (2 g/kg) suspended in 10% Tween 80.

**Figure 1.**
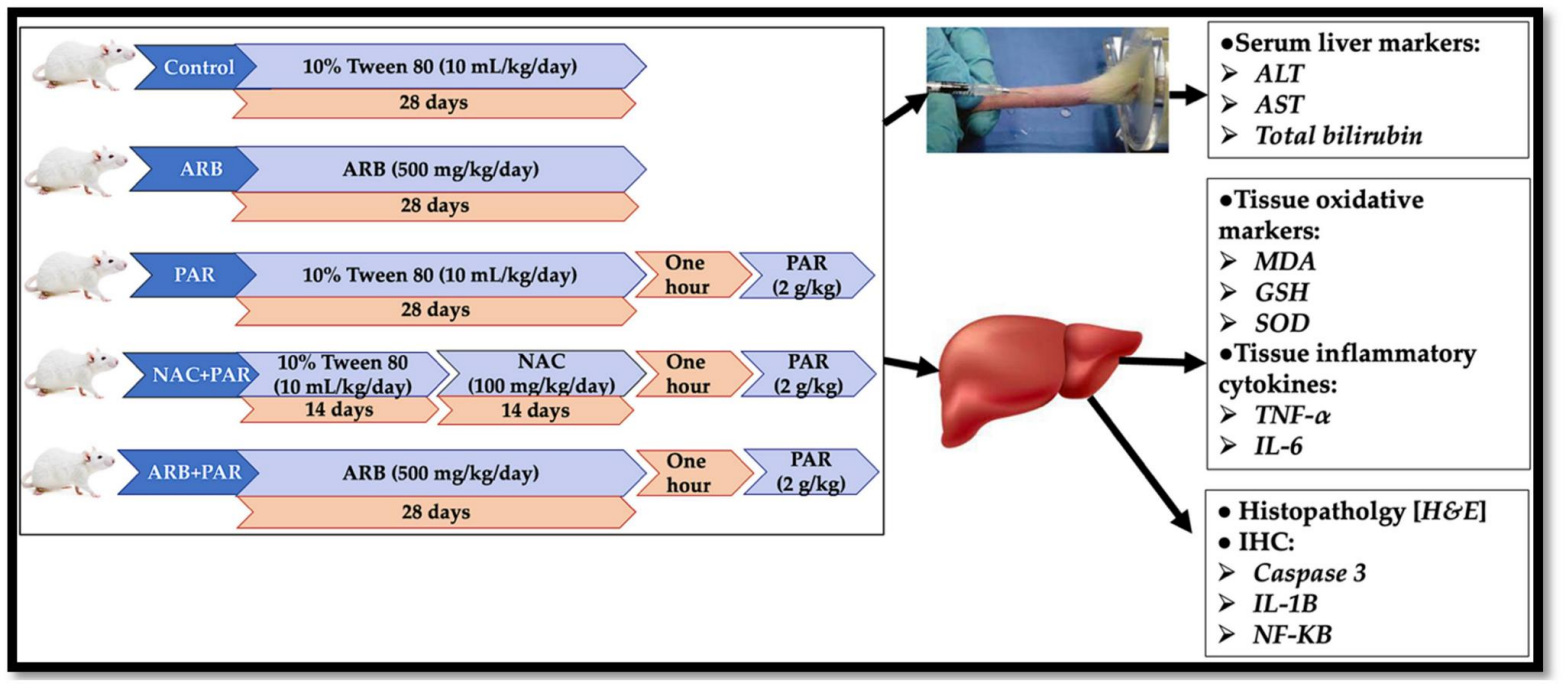
The timeline and design of the study.

In general, drug treatments were administered via oral gavage. Animals in PAR, NAC + PAR, and ARB + PAR groups received PAR as a single dose one hour after the last dose of Tween 80, NAC, or ARB, respectively. The timeline and design of the study is illustrated in Figure 1.

#### Euthanasia and tissue sampling

Blood was collected from rats’ tail veins 4 h after PAR intoxication, then serum samples were separated by centrifugation at 3000 rpm for 5 min and stored at −20 °C for biochemical analysis. Animals fasted for 24 h after PAR intoxication, euthanized by ketamine and xylazine anaesthesia followed by cervical dislocation. A laparotomy was done, and the livers were rapidly removed, washed in ice-cold saline solution, and divided into two subsets. The first part was homogenised in phosphate buffer saline (0.1 M PBS, pH 7.4) and centrifuged at 10,000 rpm for 30 min at 4 °C, and supernatants were stored at −80 °C until biochemical assays. The second part of the liver was processed for histopathological study.

#### Assessing the biochemical parameters

##### Assessment of liver function

The levels of aspartate aminotransferase (AST), alanine aminotransferase (ALT), and total bilirubin were estimated in serum using commercial kits following the manufacturer’s instructions, as detailed in Table 1. The results were recorded and analysed at 505 nm using a UV-visible spectrophotometer.

**Table 1.** The specifications of commercial ELIZA kits used in the biochemical parameters.

Kit	Catalog#	Function	Source
**AST**	AS 10 61 (45)	Liver function biomarkers	Biodiagnostic, Giza, Egypt
**ALT**	AL 10 31 (45)		
**Total bilirubin**	BR 1111		
**MDA**	MD2529	Oxidative marker	
**GSH**	GR2511	Antioxidant markers	
**SOD**	SD2521		
**mtROS**	LS-F9759		LS Bio, Seattle, USA
**NO**	MBS723386		MyBioSource, USA
**TNF-α**	MBS2507393	Proinflammatory markers	
**IL-6**	MBS269892		

##### Assessment of oxidative stress biomarkers

Liver tissue levels of malondialdehyde (MDA), reduced glutathione (GSH), and Superoxide dismutase (SOD) activities were assessed calorimetrically using commercial kits, while mtROS and NO levels were measured using the ELISA technique following the manufacturer’s instructions as detailed in Table 1.

##### Assessment of proinflammatory cytokines

The levels of tumour necrosis factor-α (TNF-α) and interleukin-6 (IL-6) in liver tissue were evaluated using ELISA kits following the manufacturer’s instructions, as detailed in Table 1.

#### Histopathological examination

Fresh portions from liver tissue were fixed in 10% NBF at room temperature for 24 h, dehydrated in ascending concentrations of ethanol, inserted in melted paraffin wax, sectioned at 4–5 μm thickness, and finally stained with haematoxylin and eosin.^43^ The sections were examined for histopathological changes and photographed using ordinary light microscopy. A histopathologic scoring for PAR-induced liver injury was adapted per Kleiner and colleagues.^44^

#### .*Immunohistochemistry*

The immunoperoxidase method was used to explore caspase 3, interleukin 1B (IL-1B), and nuclear factor-kappa beta (NF-kB) expressions in liver tissue. In brief, three μm thick sections were deparaffinized and blocked for endogenous peroxidase activity. After antigen retrieval, sections were left to cool for 60 min, then incubated with primary antibodies at 4 °C overnight, as detailed in Table 2. Then, sections were washed twice with PBS, incubated with labelled secondary antibodies, and further counterstained with Mayer’s haematoxylin. In the end, sections were dehydrated, xylene cleared, and covered with glass covers to be examined microscopically.^43^^,^^45^

**Table 2.** Primary antibodies applied for immunohistochemistry.

Antibody	Caspase 3	IL-1B	NF-KB
**Catalog#**	PDR172	Ab283818	E-AB-32232
**Host and type**	Rabbit, polyclonal, conjugated	Rabbit, recombinant multiclonal	Rabbit, polyclonal, unconjugated
**Optimal dilution**	Optimized for use	1:500	1:200
**Cellular localisation**	Cytoplasmic	Cytoplasmic	Nuclear
**Function**	Proapoptotic	Proinflammatory	Proinflammatory, Proapoptotic

#### Morphometric analysis

Analysis of Caspase 3, IL-1B, and NF-KB expression in the immunostained sections was accomplished by measuring the mean area% in the six most representative randomly selected non-overlapping fields in each section at magnification x400. Sections were snapped using a digital camera (Leica ICC50, 5.0 megapixels). Morphometric analysis was done via Leica Qwin-500 LTD-software image analysis computer system Ltd. (Cambridge, England).

### In-silico studies

In the current study, AutoDock Vina 1.1.2 software^46^^,^^47^ was used to conduct the molecular docking study, and MGL Tools 1.5.7 was implemented to prepare the protein and the ligand and save them in pdbqt format, which is a pre-requisite to carrying out the docking procedure by Autodock Vina. The results are visualised by Discovery Studio Visualiser v21.1.0.20298.^48^

The target enzymes’ pdb files were downloaded from the protein data bank (Berman et al., n.d.) with the following IDs: 3ETR (xanthine oxidase), 2OYE (cyclooxygenase-1), 6NCF (lipooxygenase), and 1E7U (PI3K). The co-crystallized ligand in each protein file was used to identify the pharmacophoric features of the tested enzymes and their binding sites.

### Statistical analysis

The data and measures obtained were analysed using the GraphPad Prism software, version 5 (Inc., San Diego, USA), and the data were presented as mean ± standard deviation. One-way ANOVA, followed by post-hoc Tukey tests, was utilised for intergroup comparison. Statistical significance was set at *p* < 0.0001.

## Results and discussion

### Phenolics and flavonoids analysis of ARB fruit extract

For the first time, the phenolics and flavonoids of the *A. pavarii* fruits were detected by LC-MS/MS and measured by quantitative spectrophotometric assays. Our findings indicated the presence of 354.54 mg/g and 36.2 mg/g of the phenolics and flavonoids as equivalents to gallic acid (standard phenolic acid) and rutin (standard flavonol glycoside), respectively (Table 3). The phenolics and flavonoids of the plant extract from stem and leaf parts have been determined by Buzgaia et al.,^26^and revealed much higher quantities of these constituents compared to the current findings. In addition, the phenolics and flavonoids of A. pavarii aerial parts have been measured by Ezzat et al.^49^ and indicated a lower level of phenolics (163.6 mg gallic acid/g) and a higher level of flavonoids (206.1 mg rutin/g) compared to the current measurements for these constituents (Table 3). Therefore, our results and previous reports indicated variations in the phenolic and flavonoid concentrations in different plant sections, e.g., fruits, leaves, stems, and aerial parts. The current findings and reported quantities of phenolics and flavonoids also indicated the potential of the plant as a source for these important secondary metabolites from the perspective that the plant is edible and used by local people in traditional medicine.

**Table 3.** Quantitative constituents and antioxidant measurements of *A. pavarii* fruit extract.

Quantitative tests	TPC^a^	TFC^b^	DPPH^c^	FRAB^c^	ORAC^c^
**Results**	354.54 ± 0.79	36.2 ± 1.90	37.7 ± 1.23	27.10 ± 0.12	13.32 ± 1.45

^a^ Total phenolic content in mg/g gallic acid equivalent of the fruits extract;

^b^ Total flavonoid content in mg/g rutin equivalent of the fruits extract;

^c^ Results of the DPPH (2,2-Diphenyl-1-picrylhydrazyl), FRAB (ferric ion reducing antioxidant power), and ORAC (oxygen radical absorbance capacity) in mg Trolox equivalents. All the results were displayed as the mean ± standard deviation of three independent experiments.

The present work also includes LC-MS/MS analysis of the *A. pavarii* fruit extract. The results indicated the presence of 35 compounds of phenolic acids and flavonoids in nature. The compounds have been identified based on the available mass spectral data from NIST (the National Institute of Standards and Technology) and by comparing the mass fragmentation pattern with those reported in the literature (Table 4). In addition to the previous identification methods, the identity of some compounds (assigned by asterisks (*) in Table 4) has been proved by matching their retention time (RT) with the RT of standard phenolic acid and flavonoid standards. The abundance of the identified compounds in the A. pavarii fruit extract was calculated relative to the total peak areas in the LC-chromatogram and indicated that the identified compounds represented 11.397% of the compounds in the chromatogram (Table 4). The results demonstrated in Table 4 indicated the presence of 13 phenolic acid-based compounds, which represented a relative abundance of 1.95%. Furthermore, gallic acid (relative abundance of 0.78%) and its derivatives, i.e., galloyl quinic acid (0.05%), galloyl shikimic acid (0.12%), epigallocatechin (0.24%), trigalloyl glucoside (0.05%), and methyl gallate (0.68%), were found to be the most abundant phenolic acids in the *A. pavarii* fruit extract.

Several flavonoids have also been identified in the form of aglycones and their glycosides, including the procyanidins in the plant extract. The total relative abundance of the identified flavonoids was calculated at 9.14%. Among them, the procyanidins, which were reported in other parts of the plant, e.g., leaves and stems^,^^26^ were found to be the most abundant constituents in the plant (4.79%).

The HPLC matching analysis with specific phenolic and flavonoid standards confirmed the presence of two gallic acid-based compounds, i.e., galloyl shikimic acid and digalloyl shikimic acid, and indicated the dominancy of the flavonol-based glycosidic compounds such as myricetin rhamnoside, quercetin-3-*O*-glucoside, quercetin 3-*O*- rutinoside, and quercetin 3-*O*-rhamnoside. Furthermore, catechin and its dimer and trimer procyanidin-based compounds were also identified in the extract of the *A. pavarii* fruits, aiding the corresponding authentic standards.

The mass fragmentation pattern has been used as a tentative-based identification tool, especially for glycosylated and corresponding non-glycosylated phenolic acids and flavonoids. For example, the mass fragment spectra of gallic acid glucoside and trigalloyl glucoside showed molecular ion peaks at 331.0640 and 635.0840 [M-H]-, respectively, and several atomic mass units (AMU), including the 169 AMU [M-glu]- for the aglycone, gallic acid (the mass spectra **of the identified compounds are available in the supplementary file)**. The protocatechuic acid O-hexoside mass spectrum was demonstrated in the [M-H]- base peak at m/z 315.0698 and the AMU fragment at 108, which was assigned for the aglycone protocatechuic acid mass unit [M-H-glu]-. The same manner of mass spectral analysis was used in the identification of flavonoid glycosides. For example, all the quercetin-based glycosylated flavonoids, e.g., quercetin 3-O-rutinoside, quercetin-O-galloyl-glucoside, quercetin-3-O-glucoside, and quercetin 3-O-rhamnoside, were identified by the presence of their molecular ion peaks [M-H]- (m/z 609.1396, 615.0919, 463.0833, 447.0884, respectively) and the presence of MAU 300 [M-2H-glu]- or 301 [M-H-glu]- in their mass fragmentation spectra. Furthermore, the mass spectra of luteolin-7-glucoside and myricetin rhamnoside were also showed the molecular ion peaks [M-H]- at m/z 447.0904 and 463.0821 and the MAU of the aglycones luteolin and myricetin at m/z 285 and 317 [M-H-glu]-, respectively.

The phenolic and flavonoid constituents of the different plant parts rather than fruits have been investigated in previous reports, which supported the current analysis’s findings. The plant aerial parts have been analysed by HPLC and revealed the presence of several phenolic acids and flavonoids, and gallic acid, chlorogenic acid, protocatechuic acid, and rutin have been identified as the major constituents of the plant aerial parts.^50^ The LC-MS analysis of the leaf part of the plant has also been investigated and revealed the dominance of gallic acid-based phenolic acid derivatives such as gallic acid hexoside and galloylquinic acid, in addition to the flavanol- and catechin-based flavonoid derivatives.^51^ Similar compounds have also been detected in the stem part of the plant.^26^

The hepatoprotective effect of polyphenols has often been associated with their antioxidant activity.^52–54^ For example, quercetin is a very efficient antioxidant in many diseases such as cancer, cardiovascular disease, and neurodegenerative disorders.^55^^,^^56^ As shown, the derivatives of this compound were detected in the studied fruit.

A range of health-related properties are also reported for catechin (flavan-3-ols) and procyanidin dimers and trimers, such as antiviral, insulin-like, antitumor, anti-inflammatory, and antioxidant activities.^57^^,^^58^

### In vitro antioxidant activity of A. pavarii fruit extract

The results demonstrated in Table 3 showed that the *A. pavarii* fruit exhibited remarkable reducing characteristics towards the ferric ion measured by the FRAP assay at 27.10 mg of Trolox equivalent per gram of the plant. Meanwhile, the ORAC result revealed that the methanolic extract of the fruit displayed a noticeable antioxidant capacity of 13.32 mg Trolox equivalent. Furthermore, the fruit extract also exhibited scavenging activity for the free radical DPPH, measured at 37.7 Trolox equivalent. The phenolic acids and flavonoids of the *A. pavarii* fruit extract played a major role in the plant antioxidant activity and the current findings of the reducing and free radical scavenging activities of the plant extract. Part of the antioxidant activity of *A. pavarii* fruits might also be attributed to their vitamin C and carotenoids, which have been reported in the fruits of the plant.^59^

### Hepatoprotective and in vivo antioxidant activities of ARB fruit extract

#### ARB fruit extract restored PAR-induced abnormality in liver function parameters

**Figure 2.**
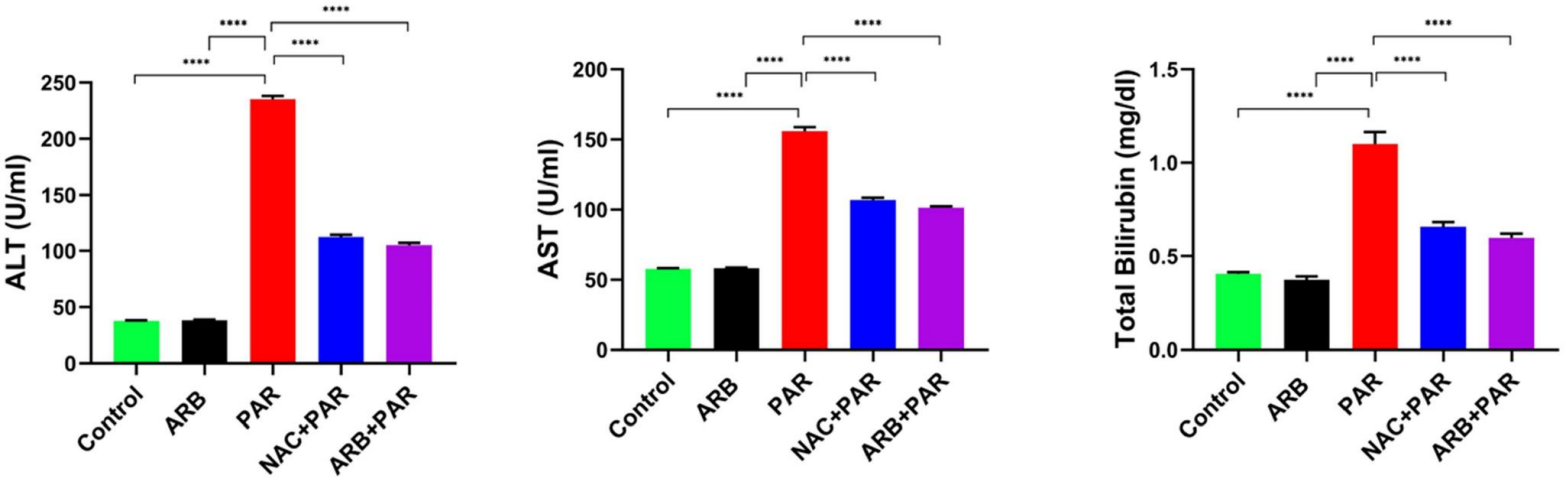
Effects of ARB extract on serum liver function markers in rats subjected to PAR-induced liver toxicity. Data are represented as mean ± SD (*n* = 6) using one-way ANOVA followed by Tukey’s multiple comparison test at ****P < 0.0001.

As shown in Figure 2, the results of the liver function parameters indicated that PAR administration led to acute liver damage, demonstrated by a significant increase in the ALT, AST, and total bilirubin levels by 6.4-, 2.7-, and 2.8-folds, respectively (*p* < 0.0001). However, pre-treatment with ARB extract attenuated PAR-induced hepatotoxicity by significantly reducing ALT, AST, and total bilirubin levels by 2.3-, 1.5-, and 2-fold, respectively, compared to the PAR group (*p* < 0.0001). It is noteworthy that ARB alone or NAC + PAR had a similar effect as control and ARB + PAR groups, with no substantial distinction between them. The liver function restoring property of ARB fruit is almost attributed to the fruit’s phenolic and flavonoid contents, which are known for their potential hepatoprotective and antioxidant effects.^60^^,^^61^

#### ARB fruit extract restored the disrupted redox balance induced by PAR

**Figure 3.**
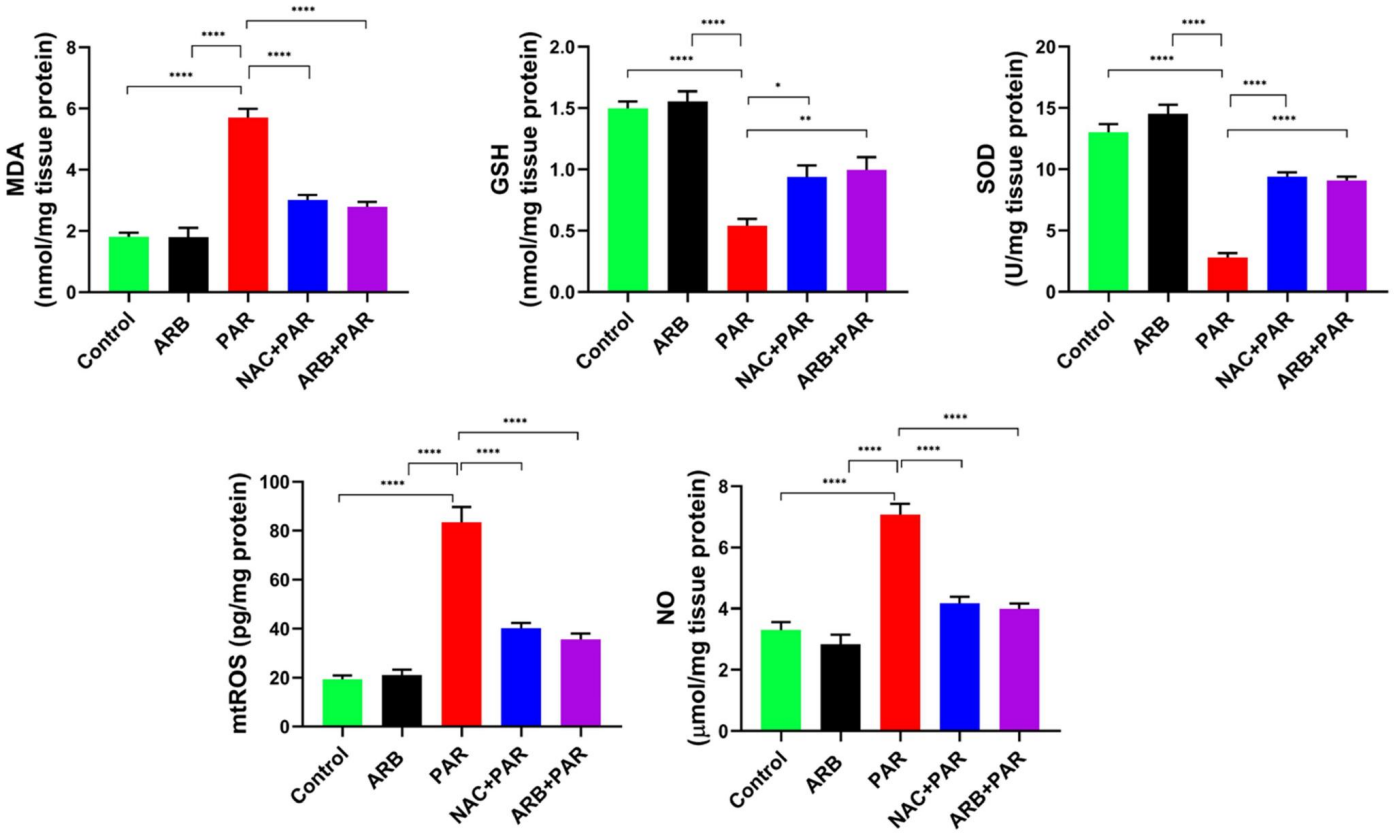
Effects of ARB extract on oxidative stress markers in rats subjected to PAR-induced liver toxicity. Data are represented as mean ± SD (*n* = 6) using one-way ANOVA followed by Tukey’s multiple comparison test at *P < 0.05, **P < 0.01, ***P < 0.001, ****P < 0.0001.

Redox balance in the hepatocyte is an essential process for liver health. The ROS produced in the hepatocyte mitochondria and endoplasmic reticulum are usually neutralised by the endogenous antioxidant system in the liver, which involves several enzymatic and non-enzymatic entities.^62^ In the current study, PAR administration induced hepatic oxidative stress damage, as shown in Figure 3, evidenced by an eminent increment in MDA, mtROS, and NO levels by 2.9-, 4.3-, and 2-folds, respectively (*p* < 0.0001), while decreasing GSH level and SOD activity by 2.9 and 5 folds, respectively (*p* < 0.0001), compared to the control group. Interestingly, the pre-treatment of animals with ARB mitigated the detrimental effects of PAR on the liver’s redox balance by reducing MDA mtROS and NO levels significantly by 2.6-, 3.2-, 1.8-folds, respectively (*p* < 0.0001) while increasing GSH levels and SOD activity by 2 (*p* < 0.01) and 2.3 folds (*p* < 0.0001), respectively, compared to the PAR group. Notably, the ARB alone and NAC + PAR groups showed no significant differences in oxidative stress-related biomarkers compared to the control and ARB + PAR groups, respectively. The restoring capacity of ARB to the protective GSH and SOD levels and its ability to MDA, mtROS, and NO reduction is mainly related to the plant contents from phenolic acids and flavonoids as an external antioxidant supplement to protect the liver against oxidative stressors drugs like PAR.

#### ARB fruit extract ameliorated PAR-induced elevation in proinflammatory cytokines in liver tissue

**Figure 4.**
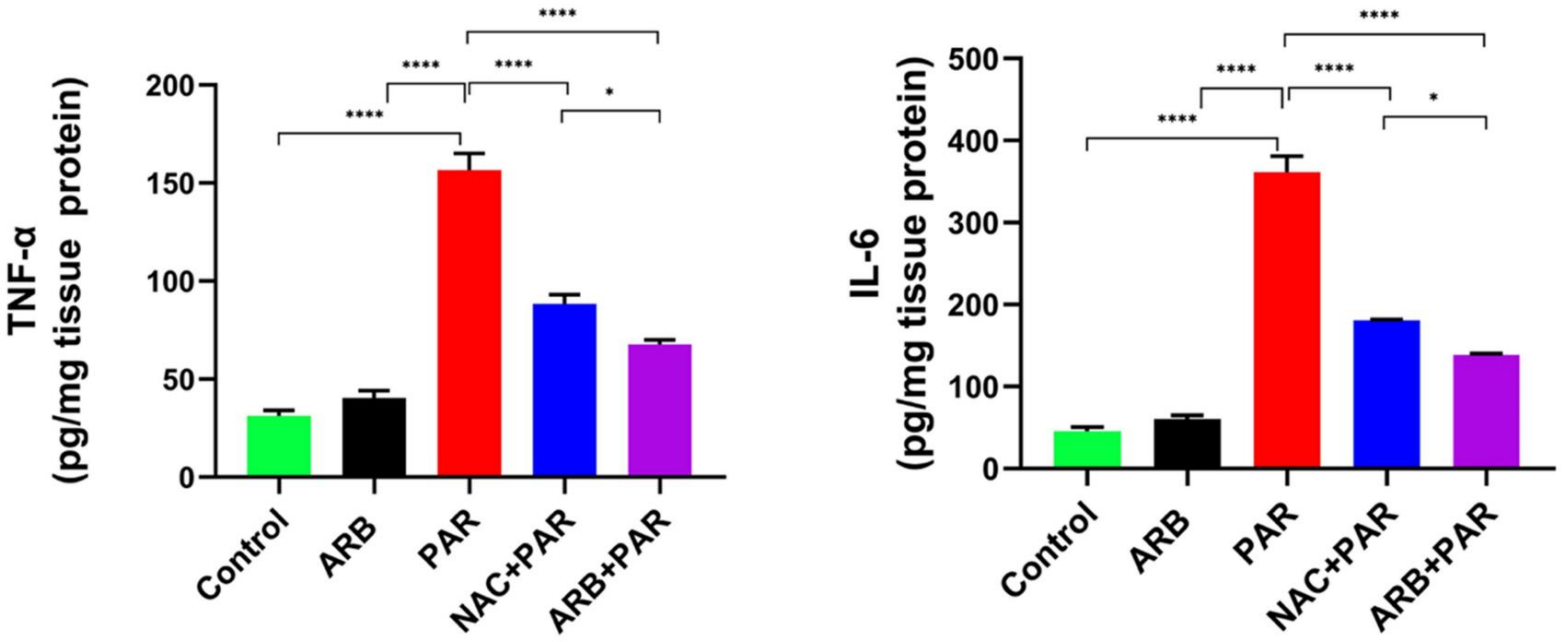
Effects of ARB extract on proinflammatory biomarkers in rats subjected to PAR-induced liver toxicity. Data are represented as mean ± SD (*n* = 6) using one-way ANOVA followed by Tukey’s multiple comparison test at *P < 0.05, **P < 0.01, ***P < 0.001, ****P < 0.0001.

The higher levels of the proinflammatory cytokines, TNF-α and IL-6, have been reported in necrotic disorders, including liver failure.^63^^,^^64^ Their levels are also elevated in the hepatocytes because of the hepatic stress by different oxidative stressors such as ethanol, paracetamol, carbon tetrachloride, and others.^53^^,^^65–67^ Furthermore, these cytokines lead to liver inflammation and, subsequently, liver cirrhosis and fibrosis.^68^ As shown in Figure 4, the proinflammatory cytokines TNF-α, and IL-6, were markedly increased (*p* < 0.0001) in PAR-treated animals by 8 and 5-fold, respectively, compared to the control untreated group. The prior administration of both ARB and NAC significantly (*p* < 0.0001) alleviated the inflammation induced by PAR by decreasing the levels of TNF-α and IL-6 by 2-and 1.8-fold, respectively, compared to the PAR group. It is noteworthy that ARB administration prior to PAR intoxication exhibited an enhanced anti-inflammatory effects as it significantly (*p* < 0.05) attenuated the proinflammatory biomarkers compared to the NAC-treated group. This anti-inflammatory effect of the ARB is suggested to be attributed to the phenolic and flavonoids contents of the fruits, as the anti-inflammatory effect of these constituents has been confirmed in several reports and directly related to their antioxidant potency and their ability to restore the normal redox balance in the body.

### Histopathology results

#### ARB fruit extract amended the histopathological changes in PAR-intoxicated hepatic tissue

**Figure 5.**
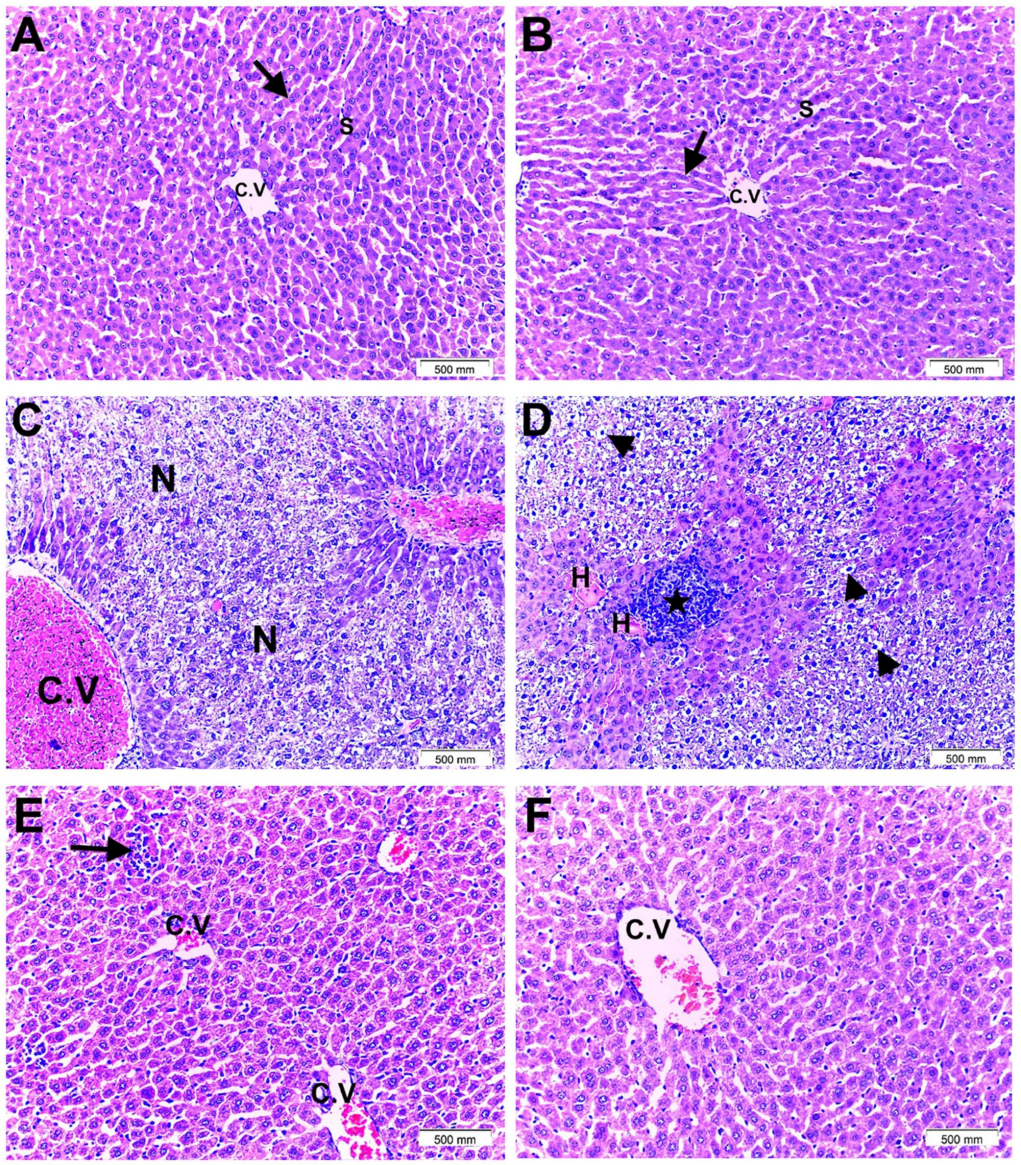
Histopathological changes in liver samples from all study groups. Haematoxylin and eosin (magnification ×200, scale bar = 500 mm). (A and B) Control and ARB groups, respectively, showed normal hepatic lobular architecture with regular cords of eosinophilic hepatocytes with vesicular nuclei (arrow) radiating from the central vein (C.V) separated by blood sinusoids (S). (C and D) PAR group revealed severely disturbed hepatic architecture with massive lobular necrosis and rarified areas (N), markedly congested central vein (C.V), focal hyaline deposits (H), heavy inflammatory infiltrates (star), and degenerated ballooned hepatocytes with pyknotic nuclei (arrowheads). (E) NAC + PAR group showed relatively improved liver histology, few inflammatory infiltrates (arrow), and mild central vein congestion (C.V). (F) ARB + PAR group revealed almost restored normal liver architecture with regularly arranged hepatocytes’ cords and mildly congested central vein (C.V).

The liver specimens obtained from PAR-intoxicated, NAC, and ARB pre-treated animals were examined microscopically for the signs of degeneration and necrosis. The control and ARB groups were similar and showed typical hepatic lobular architecture and eosinophilic hepatocytes with vesicular nuclei arranged in cords radiating from the central vein and separated by blood sinusoids (Figure 5(A,B)). The PAR group showed lobular disarray (score 1), disturbed liver architecture, massive lobular necrosis, rarified areas, congested central vein, focally deposited hyaline material, heavy inflammatory infiltrates (score 3), and degenerated ballooned hepatocytes with pyknotic nuclei (score 2) (Figure 5(C,D)). The NAC + PAR group showed improved hepatic histology, few inflammatory infiltrates (score 1), and mild central vein congestion (Figure 5(E)). These findings were per previous studies that reported marked lobular necrosis, hepatocytes’ degeneration, and inflammatory infiltration affecting hepatic lobules in animal model of PAR-induced hepatotoxicity and the partial ameliorating effect of NAC.^42^^,^^69^ Pre-treatment with ARB in the ARB + PAR group has restored the liver’s cytoarchitecture to an almost normal state with regularly arranged hepatocyte cords and a mildly congested central vein (Figure 5(F)). The scoring criteria were as depicted in Table 5.

**Table 5.** The criteria of the histopathologic scoring of PAR-induced liver injury.

Character	Score
Lobular disarray	0 = no disarray, 1 = disarray
Lobular inflammation	1 = mild, 2 = moderate, 3 = severe
Steatosis area%	1 = mild, 2 = moderate, 3 = severe
Apoptosis	0 = less than 1/HPF 1 = 1–3/HPF 2 = more than 3/HPF

PAR is one of the most common commercially used analgesics and antipyretics known for its safe use at the medically recommended dose. PAR intoxication is extremely hazardous to the liver tissue and can lead to acute liver failure with subsequent mortality. NAC, the famous commercial antidote to PAR-induced hepatotoxicity, must be given immediately after PAR intoxication to obtain the optimal effect.^69^ Recently, a large body of research has shed light on the use of medicinal plant extracts that possess potent antioxidant potentials with subsequent putative hepatoprotective activity to replace NAC in the treatment of PAR-induced hepatotoxicity.^42^ In our present study, we nominated ARB to be a potent alternative hepatoprotective candidate due to its proven antioxidant^26^^,^^40^^,^^70^ and anti-inflammatory capabilities^70^. Here we affirm, to the best of our knowledge, that our study is the first to highlight the ameliorative hepatoprotective effect of ARB fruit extract against PAR-induced hepatotoxicity on the histologic level, which proved to be a little bit better than the commercially used NAC.

#### ARB fruit extract reduced the Immunohistochemical expression of caspase 3, IL 1B, and NF-kB in PAR-intoxicated hepatic tissue

**Figure 6.**
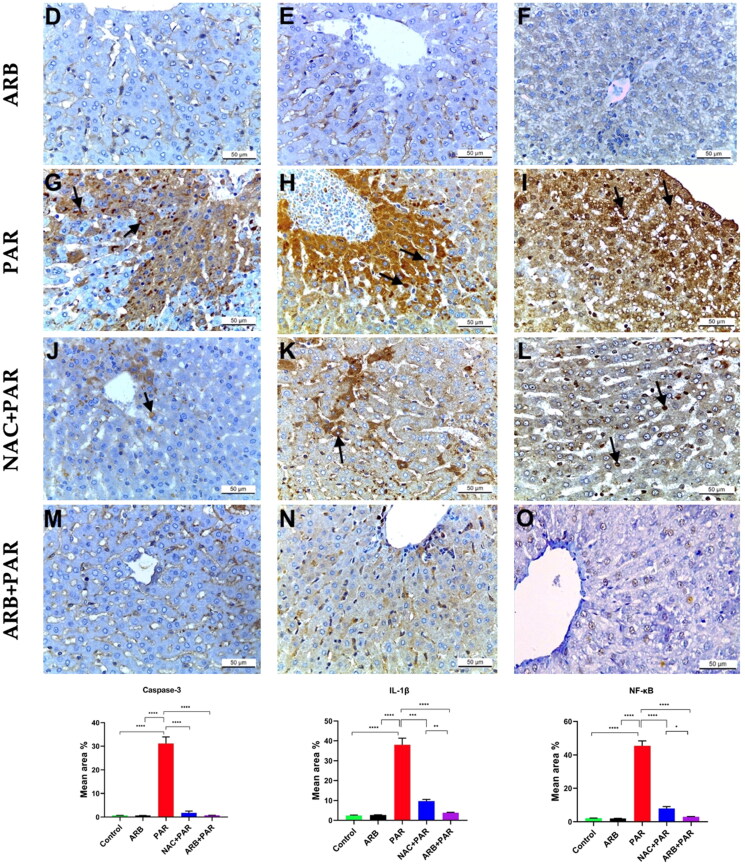
Immunohistochemical analysis of Caspase 3, IL-1B, and NF-KB in liver tissue from all study groups (magnification ×400, scale bar = 50 μm). (A-C) Control group; (D-F) ARB group; (G-I) PAR group; (J-L) NAC + PAR group; (M-O) ARB + PAR group. Control and ARB groups showed negative cytoplasmic expression of Caspase 3 and IL-1B (A,B,D,E) and negative nuclear expression of NF-KB (C,F). PAR group exhibited intense positive cytoplasmic Caspase 3 and IL-1B staining (arrows) (G,H respectively) and strong positive NF-KB nuclear expression (arrows) (I). NAC + PAR group showed mild Caspase 3 and moderate IL-1B cytoplasmic expression (arrow) (J,K respectively) and mild nuclear expression for NF-KB (arrow) (L). ARB + PAR group exhibited negative Caspase 3 and mild IL-1B cytoplasmic staining (M,N respectively) and negative nuclear expression for NF-KB (O). Data are represented as mean ± SD (*n* = 10) using one-way ANOVA followed by Tukey’s multiple comparison test at *P < 0.05, **P < 0.01, ***P < 0.001, ****P < 0.0001.

The immunohistochemical analysis of caspase 3, IL-1B, and NF-kB activities in the liver are shown in Figure 6. The control and ARB groups showed negative cytoplasmic expression of caspase 3, IL-1B, and a negative nuclear expression of NF-kB (Figure 6(A–F)). Whereas PAR intoxication imparted a strong positive cytoplasmic expression of caspase 3 and IL-1B (Figure 6(G,H)**, respectively**) and a strong positive nuclear expression of NF-KB (Figure 6(I)) that showed a statistically significant uprise at *p* < 0.0001 compared to the control and ARB groups. On the other hand, hepatocytes of NAC + PAR group showed mild positive caspase 3 and moderate IL-1B cytoplasmic expression (Figure 6(J,K)**, respectively**) and mild positive nuclear expression of NF-KB (Figure 6(L)). Pre-treatment with ARB in ARB + PAR group imparted negative caspase 3 and NF-KB reaction (Figure 6(M,O)**, respectively**) with mild positive IL-1B reaction (Figure 6(N)). Statistical analysis showed that the pre-treatment with NAC or ARB before PAR administration exhibited a statistically significant regression at *p* < 0.0001 in the immunoexpression of Caspase 3, IL-1B, and NF-KB in both NAC + PAR and ARB + PAR groups compared to the PAR group. Noteworthy that Caspase 3 expression in pre-treated groups with NAC or ARB had no substantial difference at *p* < 0.0001 compared to control and ARB alone groups, respectively. Additionally, pre-treatment with ARB in ARB + PAR group showed a significant decline in tissue expression of IL-1B (*p* < 0.01) and NF-KB (*p* < 0.05) compared to NAC + PAR group. No statistically significant differences were noticed at *p* < 0.0001 between the control and ARB groups in the immunoexpression of all measured markers.

NF-KB is a crucial mediator of cellular oxidative stress and disturbance in the liver redox system, which plays an important role in the induction of inflammation by activating cytokines and immune-responsive genes and induced hepatocyte injury and death^71^. Our findings indicated that ABR restored the levels of GSH and SOD and reduced the levels of MDA (Figure 2), the mechanism which could be involved in the reduction of PAR-related oxidative stress-induced activation of NF-KB in the liver cells. This effect will subsequently down-regulate the transcription of pro-inflammatory genes, preventing cytokine storms and DNA damage.^72^ Furthermore, liver cells viability has been reported to be affected by oxidative stress and related lipid peroxidation, which translated into the activation of caspase 3 and apoptosis. Our findings proved the in vivo antioxidant activity of ARB and its ability to restore the liver antioxidant capacity by elevating the SOD activation and GSH level and reducing the MDA level in PAR-liver intoxication. Thereby, ARB could reduce the caspase 3 activity by its antioxidant effect on injured liver cells. Further, GSH is known as a protective factor regulating the caspase 3 activity by S-glutathionylation; therefore, liver cell apoptosis and caspase 3 activity increased by reducing the levels of GSH in the hepatocytes.^71^ Thus, the restoring ability of ARB to the GSH level is a possible mechanism for the apoptosis inhibition and liver protection activity of the plant. The level of IL-1B has also been reduced by the pre-treatment of the animals by the ARB, the effect which might also be explained by the ability of the plant to enhance the redox system in the liver cells and reduce the activity of NF-KB.^73^

### .*In-silico study results*

**Figure 7.**
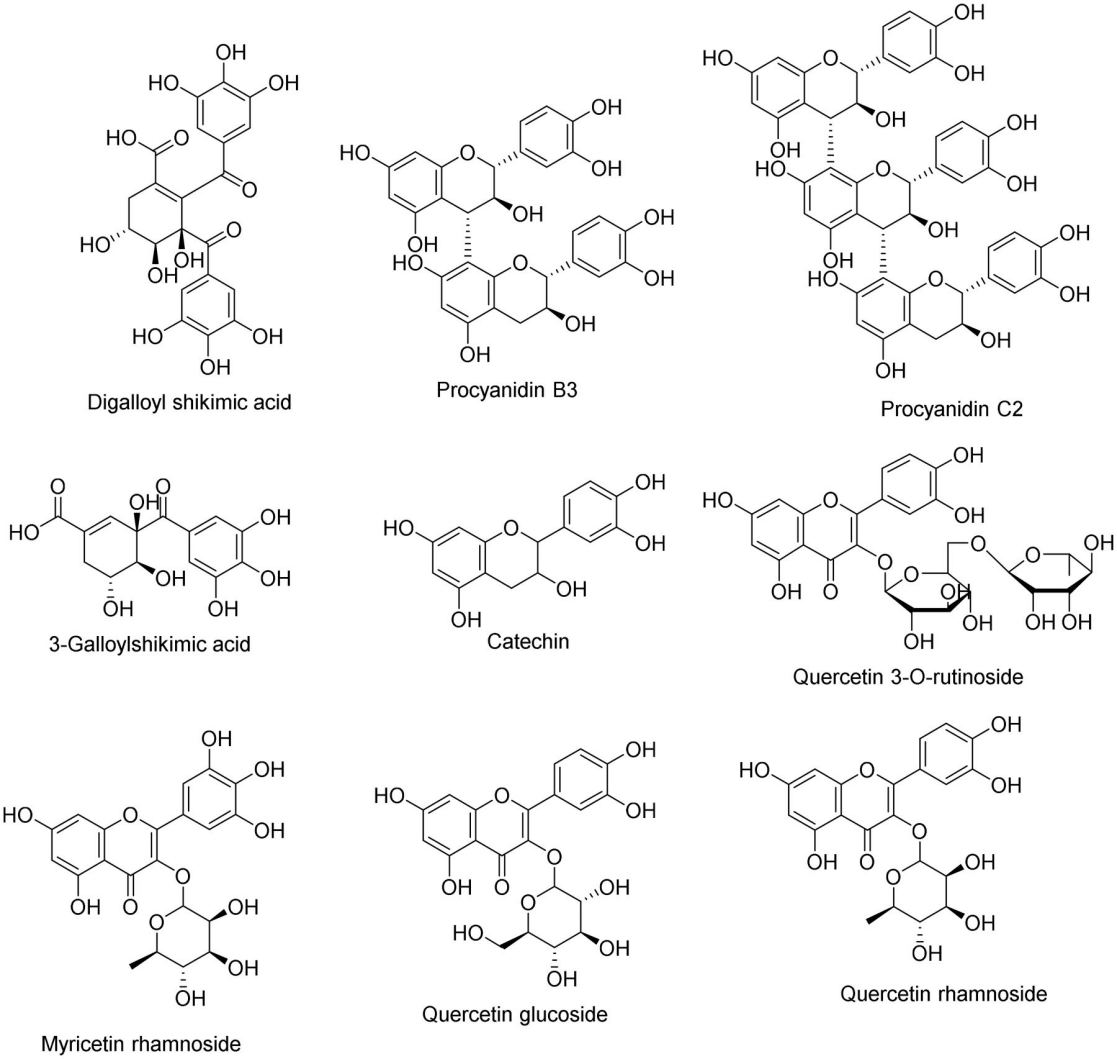
Chemical structure of the docked compounds.

Molecular docking is a shifting paradigm in drug discovery; it’s an established structure-based drug design strategy that is widely used to predict the binding affinities between the tested compound and the target protein, reducing research costs^74^. Nine compounds (Figure 7) were docked against four target proteins: xanthine oxidase (XO), cyclooxygenase-1 (COX-1), 5-lipoxygenase (5-LOX), and phosphoinositide 3-kinases (PI3K). The binding energy scores for all compounds are compared to those of the co-crystallized ligand for each protein (Table 6).

**Table 6.** Binding energy scores (kcal./mol.) for the tested compounds, the highlighted cells correspond to the two highest scores for each target.

Compound	XO	COX-1	5-LOX	PI3K
PDB ID	3ETR	2OYE	6NCF	1E7U
Co-crystallized ligand	−6.3	−8.4	−11.8	−6.5
1. Catechin	−8.4	−6.1	−6.2	−6.3
2. Procyanidin C2	−8.6	−12.4	−9.8	−12.3
3. Myricetin rhamnoside	−10.3	−9.2	−9.9	−8.3
4. Quercetin rhamnoside	−9.4	−10.2	−8.1	−7.2
5. Quercetin glucoside	−8.1	−10.1	−7.2	−7.9
6. Procyanidin B3	−11.4	−9.3	−10.5	−10.6
7. 3-Galloylshikimic acid	−8.7	−6.4	−6.5	−6.7
8. Digalloyl shikimic acid	−8.3	−7.4	−8.9	−8.6
9. Quercetin 3-O-rutinoside	−6.5	−11.6	−10.6	−9.8

#### Xanthine oxidase (XO) docking results

**Figure 8.**
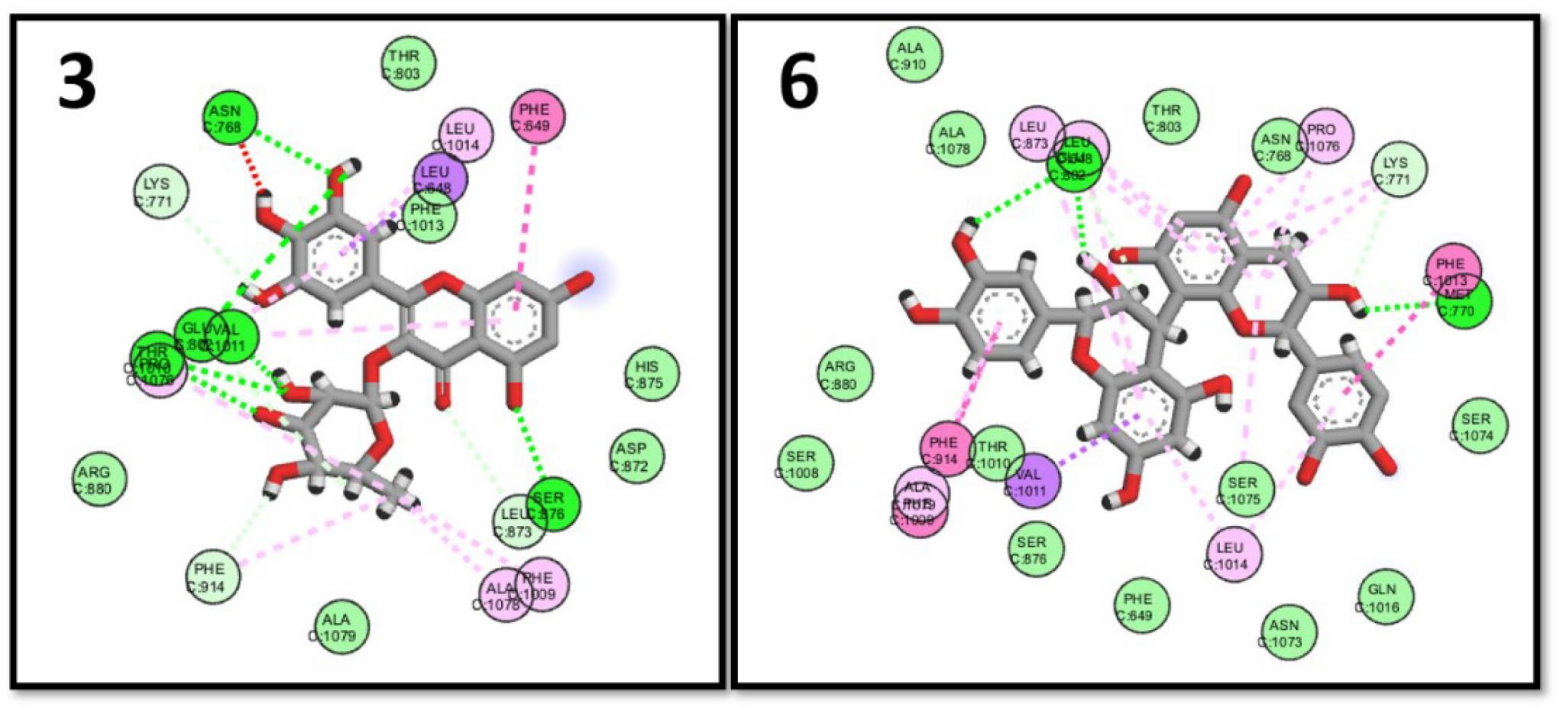
2D interactions of myricetin rhamnoside (3) and procyanidin B3 (6) in the binding pocket of XO enzyme.

The xanthine oxidase enzyme catalysed the final two steps in purine metabolism; its crystal structure (PDB ID: 3ERT)^75^ was used to perform the docking procedure. The key binding interactions with XO were through GLU802, THR1010, and VAL1011.^75^ The highest binding energy scores were observed with procyanidin B3 (6) and myricetin rhamnoside (3). The key interactions were kept in addition to several extra interactions with the neighbouring amino acids in the XO active site (Figure 8).

**Figure 9.**
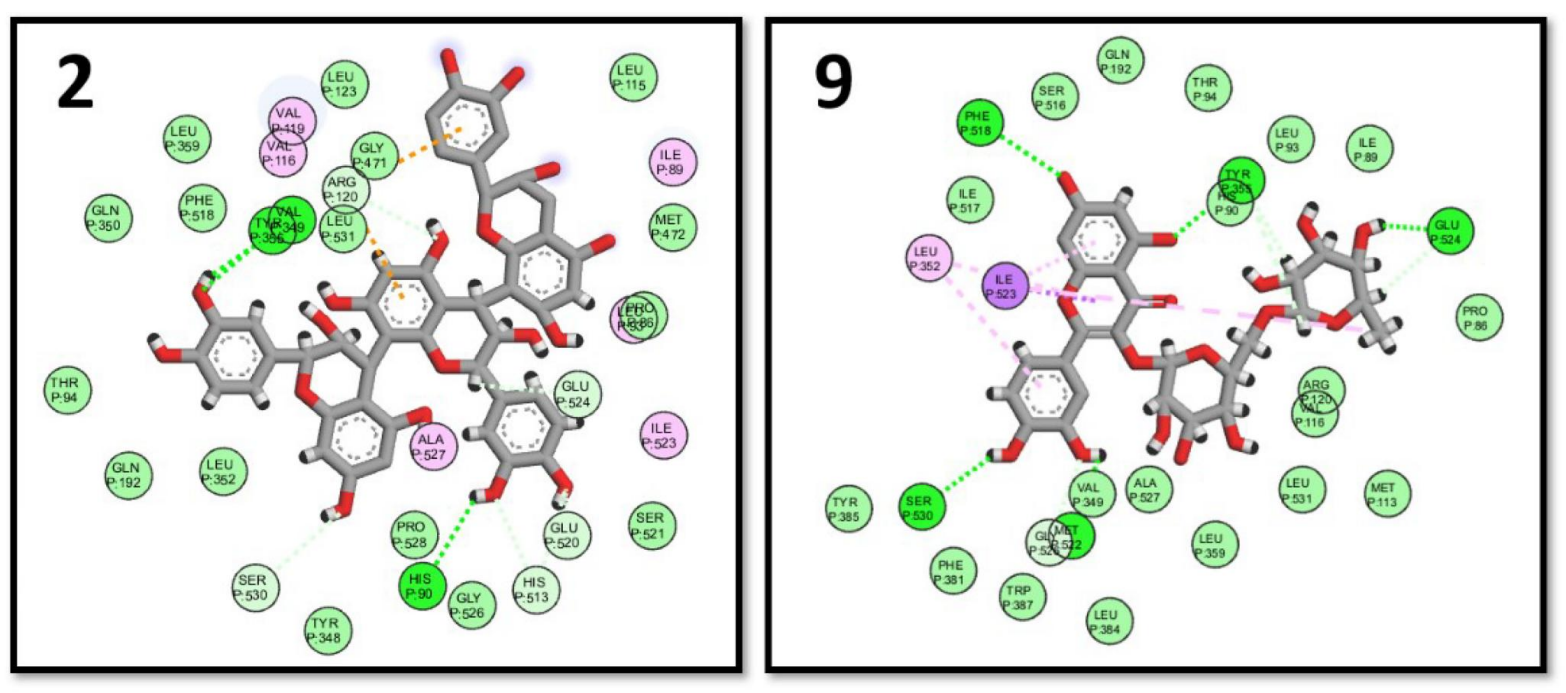
2D interactions of procyanidin C2 (2) and quercetin 3-O-rutinoside (9) within COX-1 binding pocket.

Prostaglandins, the inflammatory mediators, were biosynthesized from arachidonic acid through cyclooxygenase enzyme, PDB: 2OYE was downloaded to perform the docking study on COX-1 enzyme.^76^ Several amino acids are important to bind in the binding pocket of COX-1 enzyme as ARG120, TYR355 and ILE523 ^78^. Two of the tested compounds; procyanidin C2 (2) and quercetin 3-O-rutinoside (9) showed the highest binding scores with good binding pose with the key amino acids (Figure 9).

**Figure 10.**
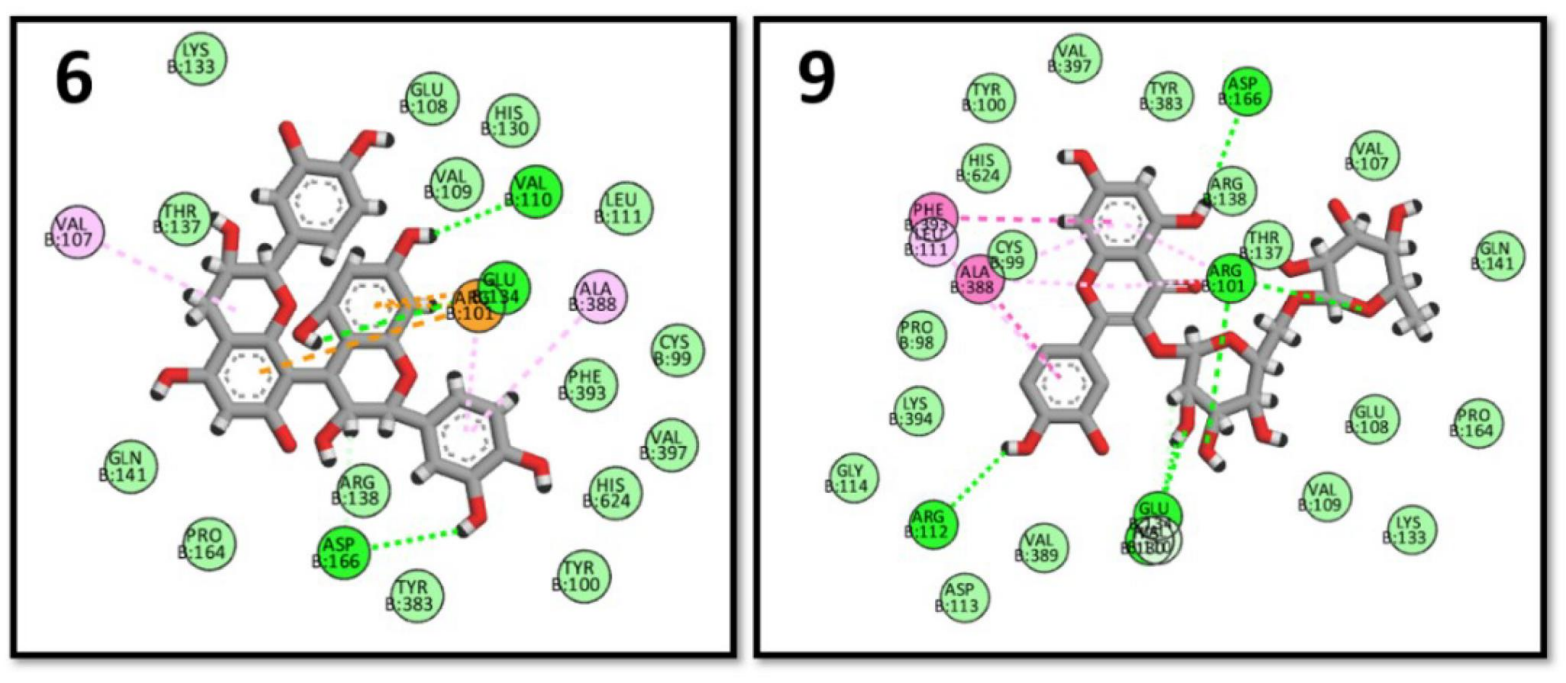
2D interactions of procyanidin B3 (6) and quercetin 3-O-rutinoside (9) within 5-LOX binding pocket.

The 5-LOX-LOXyme is responsible for the biosynthesis of leukotrienes; the inflammatory mediators, 3NCF pdb file, was used for the docking study; binding to 5-LOX active sites requires interaction with ARG101 and VAL110 amino acids.^77^ Among the tested compounds, both procyanidin B3 (6) and quercetin 3-O-rutinoside (9) exhibited the highest energy scores and showed key interactions (Figure 10).

**Figure 11.**
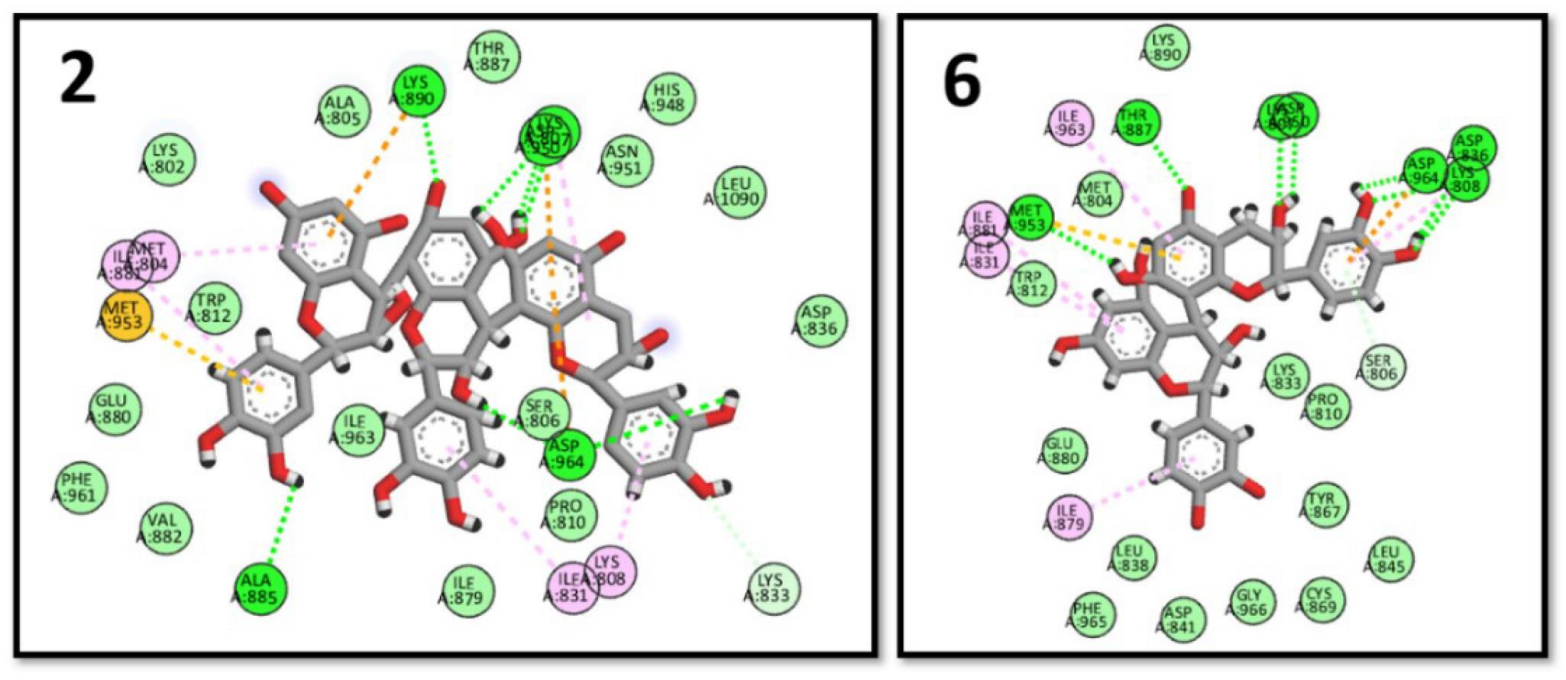
2D interactions of procyanidin C2 (2) and procyanidin B3 (6) within PI3K binding pocket.

Phosphoinositide-3-kinases (PI3Ks) regulate several key events in the inflammatory response to damage and infection. The 1E7U pdb file was used for PI3K docking.^78^ Both procyanidin C2 (2) and procyanidin B3 (6) showed the highest energy scores while maintaining the key interactions with SER806, LYS833, and ASP964 amino acid residues (Figure 11).

Finally, we can estimate that all the tested compounds have binding affinities to the target enzymes, especially procyanidin C2 (2), procyanidin B3 (6), and quercetin 3-O-rutinoside (9) which can be good candidates for future studies.

## Conclusions

In the current work, the antioxidant and hepatoprotective effects of the edible fruit, Arbutus pavarii (the Libyan Strawberry), were investigated and confirmed through a broad spectrum of biochemical and histopathological parameters in a rat model of PAR-induced hepatotoxicity besides *in vitro* antioxidant measurements of the free radical scavenging ability of the fruit extract. Several food-based phenolic acids and flavonoids were identified in the fruits of the plant, which might reflect the health benefits and safety of these fruits. The overall findings of the study indicated the potential use of the plant fruits as antioxidant supplements; however, further preclinical, and clinical investigations are needed to determine the possible side effects and food/drug interactions of ARB fruits at higher animal and human levels. In silico molecular docking studies revealed that procyanidin C2 (2), procyanidin B3 (6), and quercetin 3-O-rutinoside (9) are promising candidates to be further studied for their good binding abilities to several target enzymes involved in the inflammatory process.

## Supplementary Material

Supplemental Material
