# Supplementary material for: A multidisciplinary approach to the antioxidant and hepatoprotective activities of Arbutus pavarii Pampan fruit; in vitro and in Vivo biological evaluations, and in silico investigations
Source: J Enzyme Inhib Med Chem. 2023 Dec 28;39(1):2293639. doi: 10.1080/14756366.2023.2293639 (PMC10763860; doi:10.1080/14756366.2023.2293639)

A Multidisciplinary Approach to the Antioxidant and Hepatoprotective Activities of *Arbutus pavarii* Pampan Fruit; *In Vitro* and *In Vivo* Biological Evaluations, and *In Silico* Investigations

Fatma A. Elshibani[a], Abdullah D. Alamami[b], Hamdoon A. Mohammed[c,d], Rabab Ahmed Rasheed[e], Radwa M. El Sabban[f], Mohamed A. Yehia[g], Sherif S. Abdel Mageed[h], Taghreed A. Majrashi[i], Eslam B. Elkaeed[j], Mahmoud A. El Hassab[k,*], Wagdy M. Eldehna[l,*], Mohamed K. El-Ashrey[k,m]

[a] Department of Pharmacognosy, Faculty of Pharmacy, University of Benghazi, Benghazi, Libya
[b] Department of Basic Medical Science, Faculty of Pharmacy, University of Benghazi, Benghazi, Libya
[c] Department of Medicinal Chemistry and Pharmacognosy, College of Pharmacy, Qassim University, Qassim 51452, Saudi Arabia
[d] Department of Pharmacognosy and Medicinal Plants, Faculty of Pharmacy, Al-Azhar University, Cairo 11371, Egypt
[e] Department of Histology and Cell Biology, Faculty of Medicine, King Salman International University (KSIU), South Sinai 46511, Egypt
[f] Department of Anatomy, Faculty of Medicine, October 6 University, Giza 12573, Egypt.
[g] Department of Forensic Medicine and Clinical Toxicology, Faculty of Medicine, October 6 University, Giza 12573, Egypt
[h] Pharmacology and Toxicology Department, Faculty of Pharmacy, Badr University in Cairo (BUC), Badr city, Cairo, 11829, Egypt
[i] Department of Pharmacognosy, College of Pharmacy, King Khalid University, Asir 61421, Saudi Arabia
[j] Department of Pharmaceutical Sciences, College of Pharmacy, AlMaarefa University, Riyadh 13713, Saudi Arabia
[k] Department of Medicinal Chemistry, Faculty of Pharmacy, King Salman International University (KSIU), South Sinai 46612, Egypt
[l] Department of Pharmaceutical Chemistry, Faculty of Pharmacy, Kafrelsheikh University, Kafrelsheikh, P.O. Box 33516, Egypt
[m] Pharmaceutical Chemistry Department, Faculty of Pharmacy, Cairo University, Kasr Elini St., Cairo 11562, Egypt

***** Correspondence: mahmoud65582@pharm.tantta.edu.eg (M.A.E.), wagdy2000@gmail.com (W.M.E.).

Gallic acid glucoside

331.0640 / 3.94 (Mass/FragMass/RT/Isotope/Library/Formula/Ion Ratio)

Retention Time: 3.94 minutes
Precursor m/z : 331.0651
Fit (%) N/A **RFit (%)** N/A

Exp RT: 3.94 minutes
Analyte Name:
331.0640 / 3.94

Collision Energy = 35 ± 15 eV

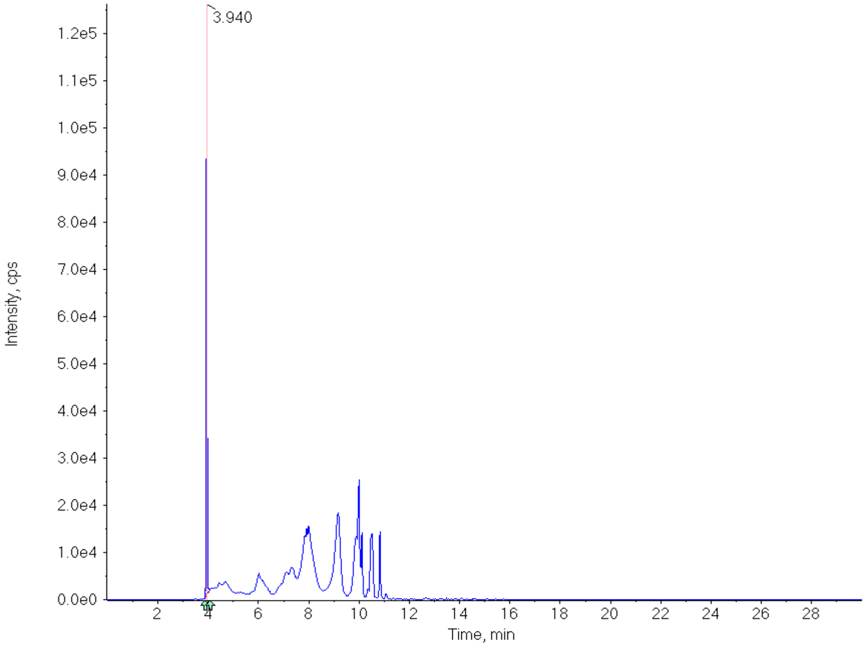

Acquired / Library MSMS

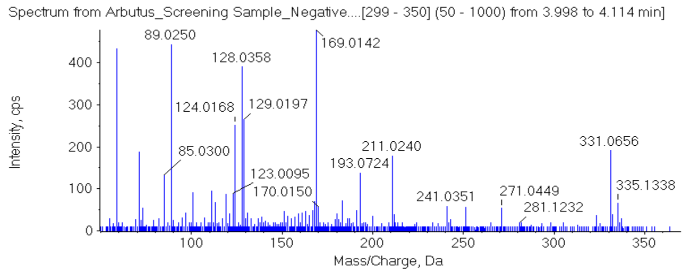

Acquired / Theoretical MS

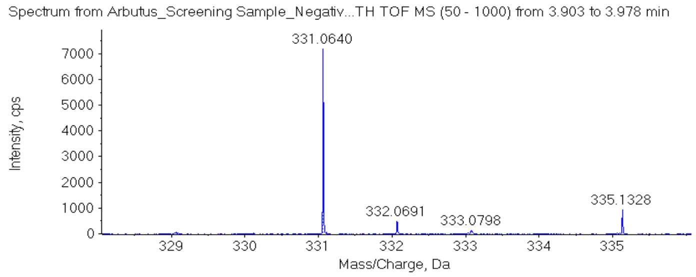

Gallic acid

169.0137 / 9.66

(Mass/FragMass/RT/Isotope/Library/Formula/Ion Ratio)

Retention Time: 9.61 minutes
Precursor m/z : 169.0148
Fit (%) 99.3% **RFit (%)** 78.0%

Exp RT: 9.66 minutes
Analyte Name:
169.0137 / 9.66

Collision Energy = 35 ± 15 eV

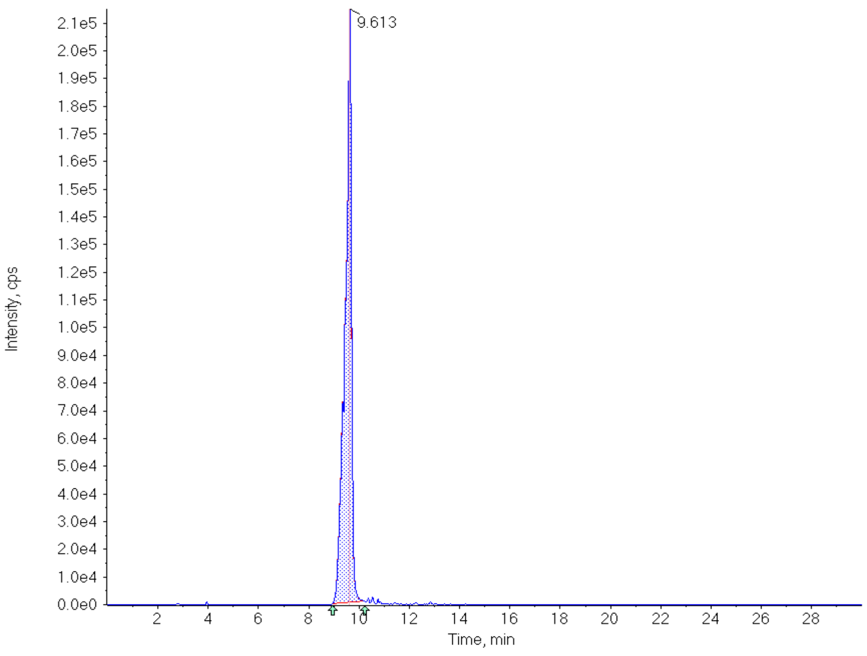

Acquired / Library MSMS

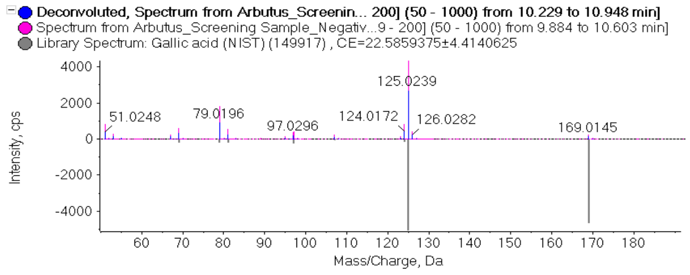

Acquired / Theoretical MS

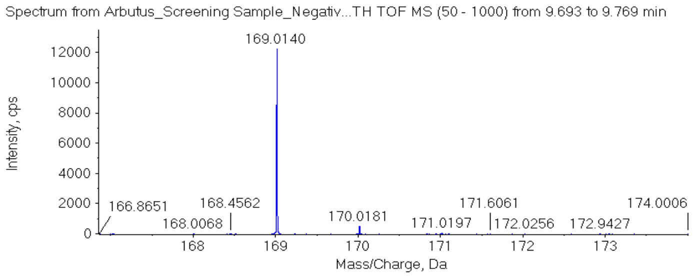

Galloyl quinic acid

343.0652 / 10.03 [M-H]- (Mass/FragMass/RT/Isotope/Library/Formula/Ion Ratio)

**Retention Time:** 10.03 minutes	**Exp RT:** 10.03 minutes
**Precursor m/z :** 343.0663	**Analyte Name:**
**Fit (%)** N/A **RFit (%)** N/A	343.0652 / 10.03 [M-H]-

Collision Energy = 35 ± 15 eV

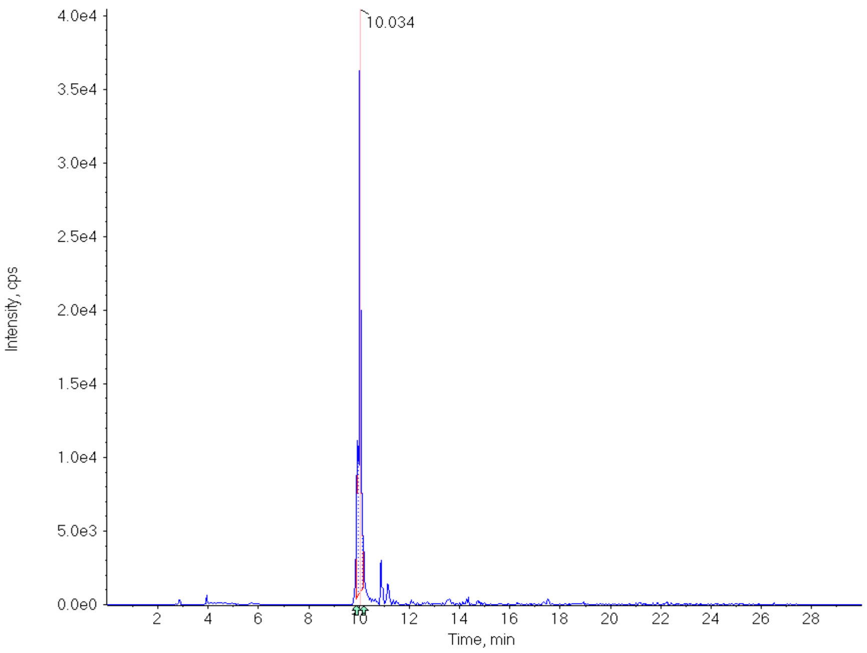

Acquired / Library MSMS

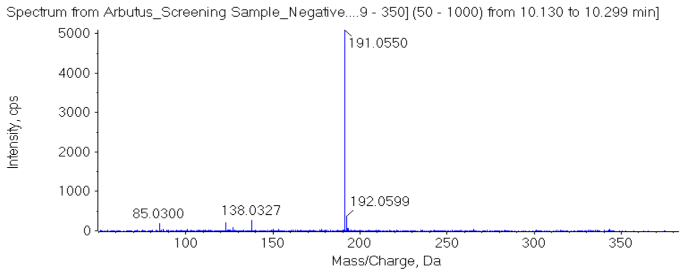

Acquired / Theoretical MS

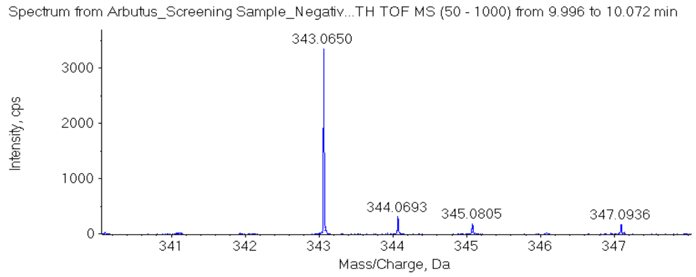

Vanillic acid-*O*-glucoside ester

329.0855 / 10.68 (Mass/FragMass/RT/Isotope/Library/Formula/Ion Ratio)

Retention Time: 10.67 minutes
Precursor m/z : 329.0866
Fit (%) N/A **RFit (%)** N/A

Exp RT: 10.68 minutes
Analyte Name:
329.0855 / 10.68

Collision Energy = 35 ± 15 eV

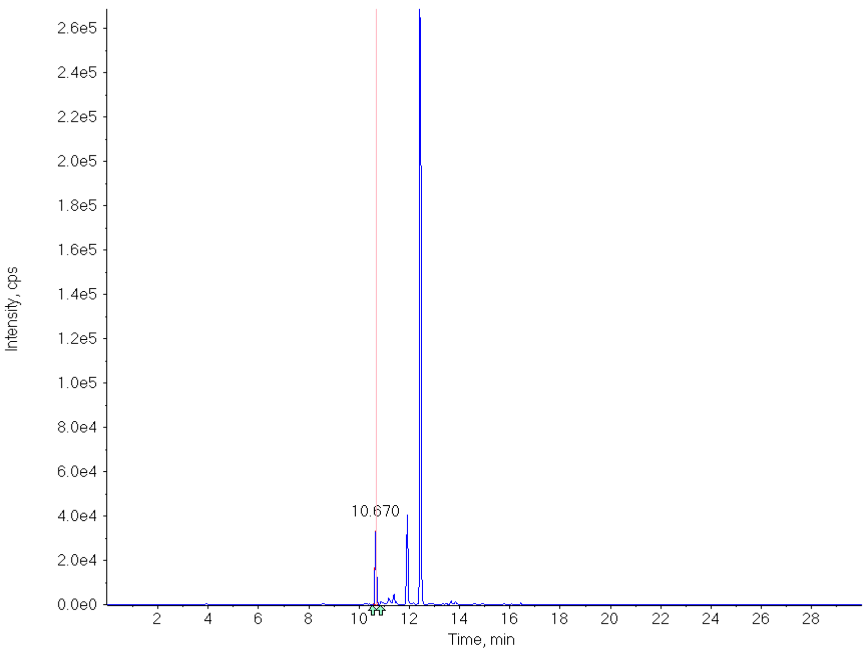

Acquired / Library MSMS

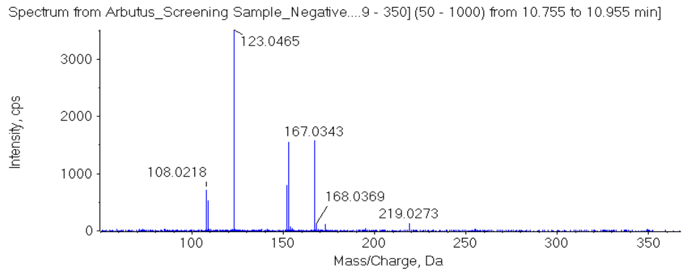

Acquired / Theoretical MS

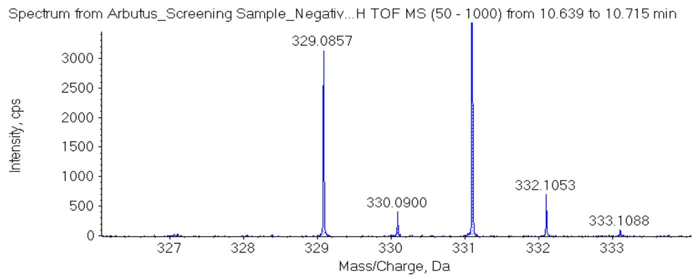

Galloyl shikimic acid

325.0550 / 10.83 (Mass/FragMass/RT/Isotope/Library/Formula/Ion Ratio)

**Retention Time:** 10.83 minutes	**Exp RT:** 10.83 minutes
**Precursor m/z :** 325.0561	**Analyte Name:**
**Fit (%)** N/A **RFit (%)** N/A	325.0550 / 10.83

Collision Energy = 35 ± 15 eV

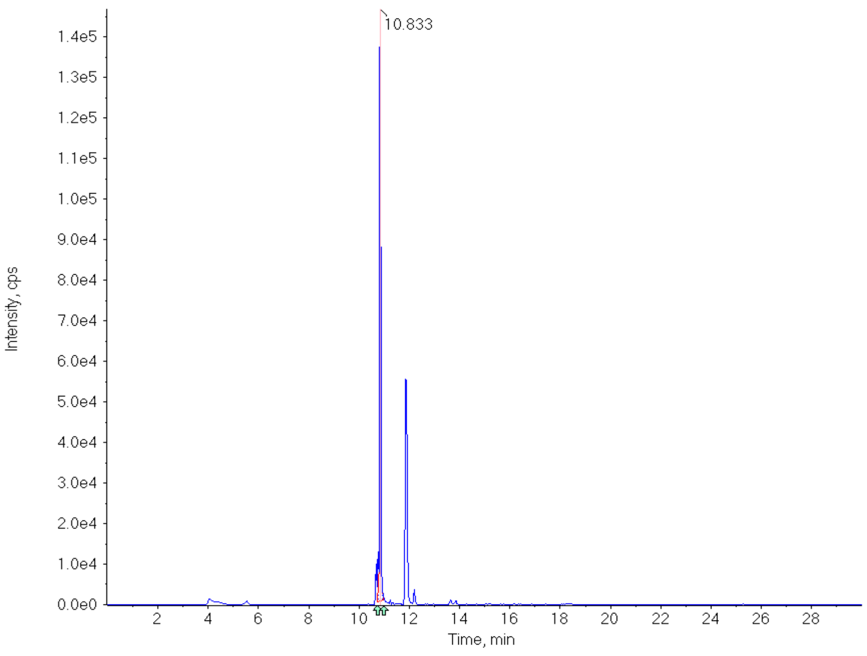

Acquired / Library MSMS

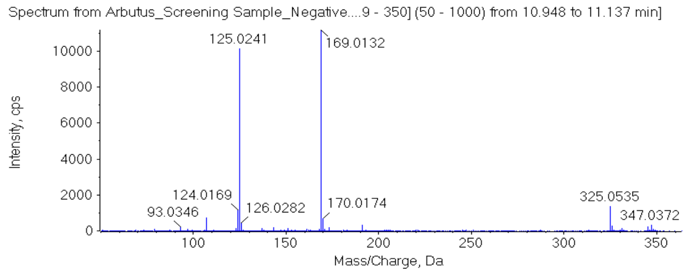

Acquired / Theoretical MS

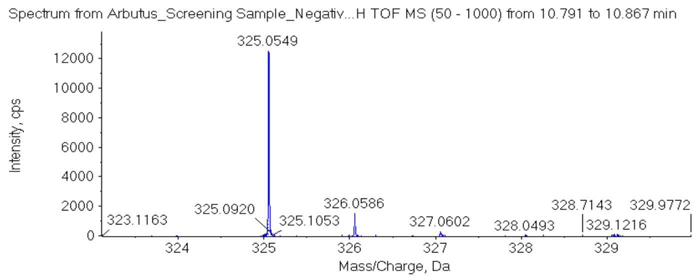

Epigallocatechin

305.0649 / 10.90

(Mass/FragMass/RT/Isotope/Library/Formula/Ion Ratio)

**Retention Time:** 10.90 minutes<br>**Precursor m/z :** 305.0660<br>**Fit (%)** 95.2% **RFit (%)** 97.1%	**Exp RT:** 10.90 minutes<br>**Analyte Name:**<br>305.0649 / 10.90

Collision Energy = 35 ± 15 eV

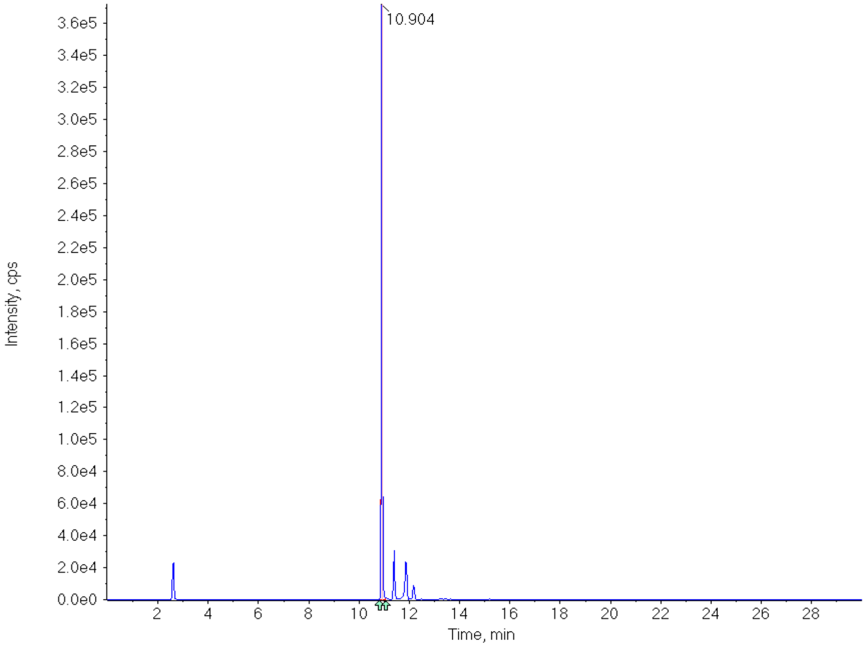

Acquired / Library MSMS

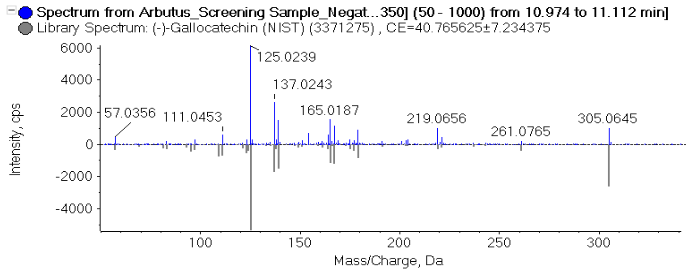

Acquired / Theoretical MS

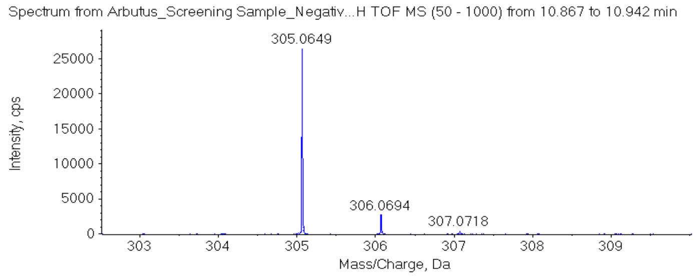

2,4-Dihydroxybenzoic acid

153.0198 / 11.28

(Mass/FragMass/RT/Isotope/Library/Formula/Ion Ratio)

**Retention Time:** 11.28 minutes<br>**Precursor m/z :** 153.0209<br>**Fit (%)** 96.2% **RFit (%)** 95.7%	**Exp RT:** 11.28 minutes<br>**Analyte Name:**<br>153.0198 / 11.28

Collision Energy = 35 ± 15 eV

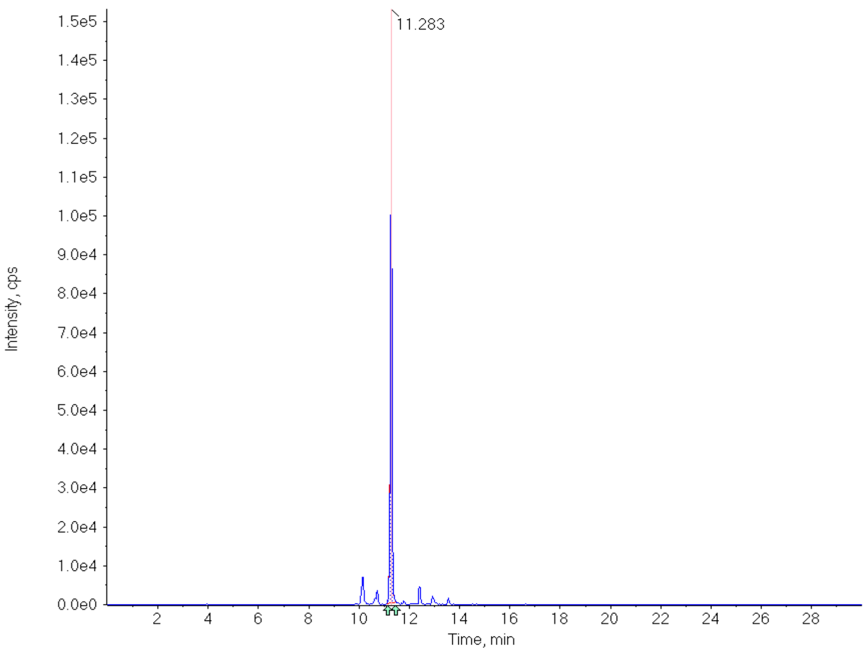

Acquired / Library MSMS

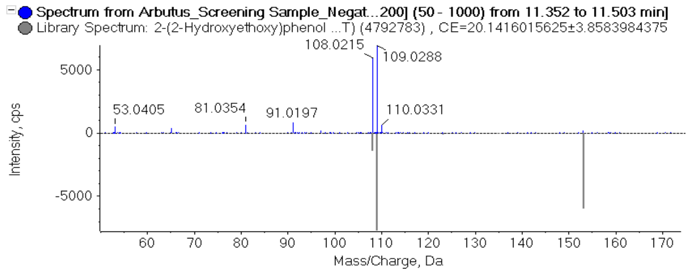

Acquired / Theoretical MS

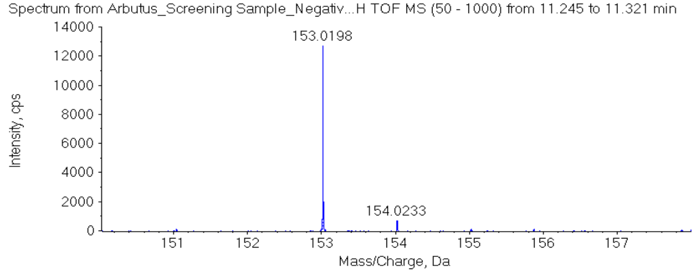

Procyanidin dimer B3

577.1272 / 11.66 [M-H]- (Mass/FragMass/RT/Isotope/Library/Formula/Ion Ratio)

Retention Time: 11.65 minutes
Precursor m/z : 577.1283
Fit (%) 98.9% **RFit (%)** 98.2%

Exp RT: 11.66 minutes
Analyte Name:
577.1272 / 11.66 [M-H]-

Collision Energy = 35 ± 15 eV

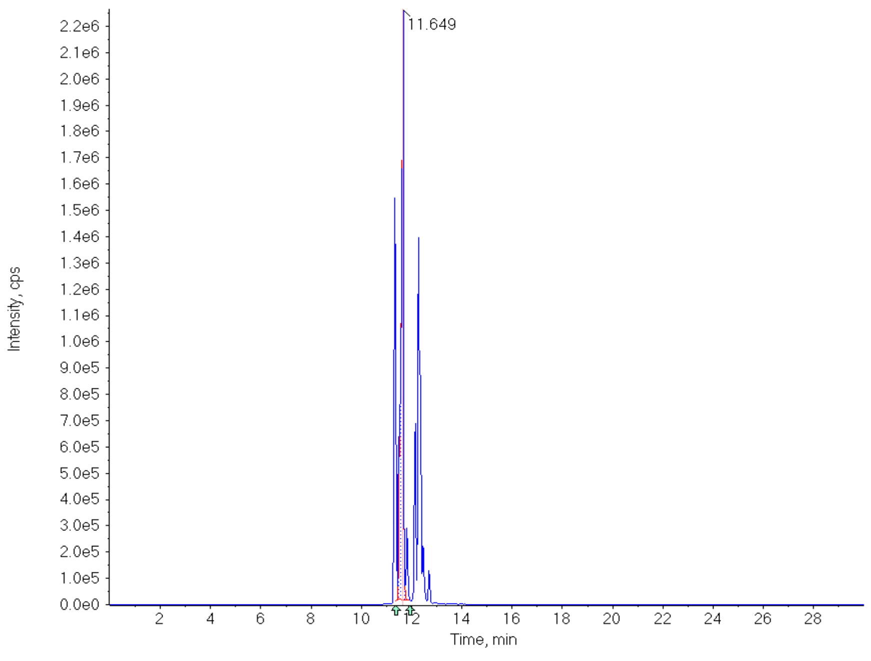

Acquired / Library MSMS

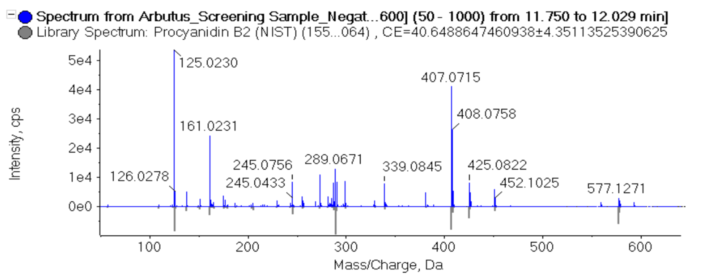

Acquired / Theoretical MS

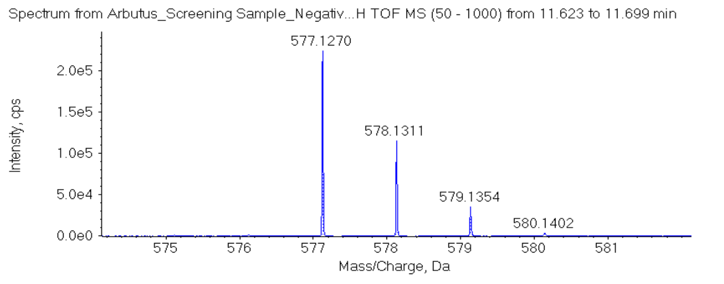

β-Type procyanidin trimer C

865.1884 / 11.66 [M-H]- (Mass/FragMass/RT/Isotope/Library/Formula/Ion Ratio)

Retention Time: 11.66 minutes
Precursor m/z : 865.1895
Fit (%) 99.4% **RFit (%)** 98.1%

Exp RT: 11.66 minutes
Analyte Name:
865.1884 / 11.66 [M-H]-

Collision Energy = 35 ± 15 eV

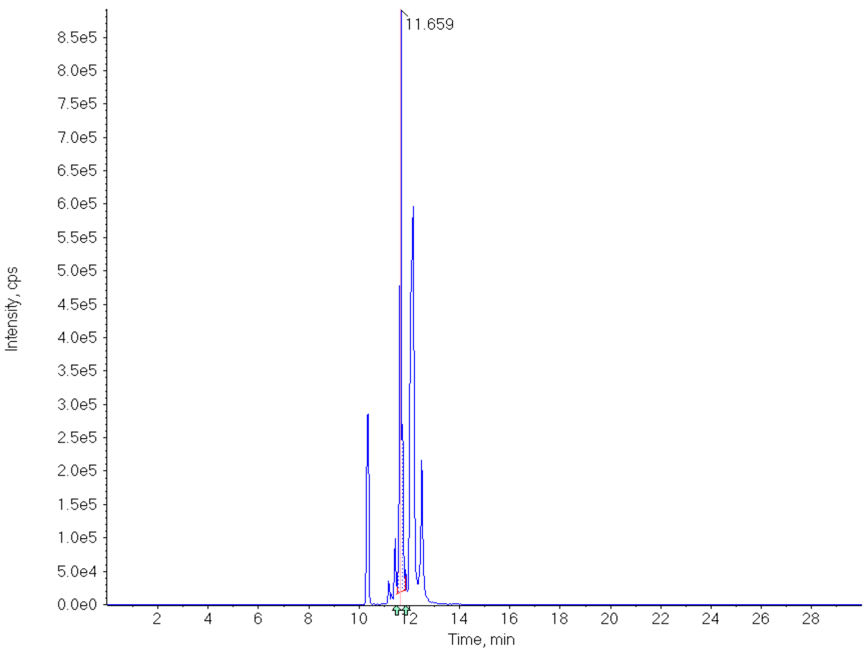

Acquired / Library MSMS

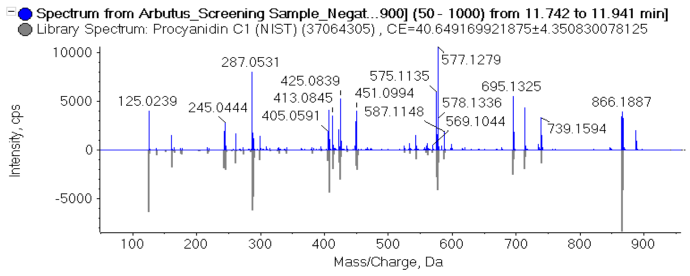

Acquired / Theoretical MS

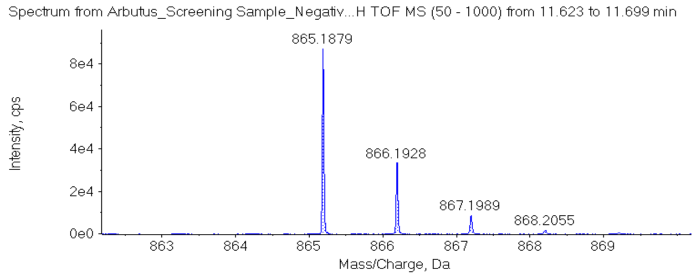

Trigalloyl glucoside

635.0840 / 12.08

(Mass/FragMass/RT/Isotope/Library/Formula/Ion Ratio)

**Retention Time:** 12.06 minutes	**Exp RT:** 12.08 minutes
**Precursor m/z :** 635.0851	**Analyte Name:**
**Fit (%)** 77.2% **RFit (%)** 99.2%	635.0840 / 12.08

Collision Energy = 35 ± 15 eV

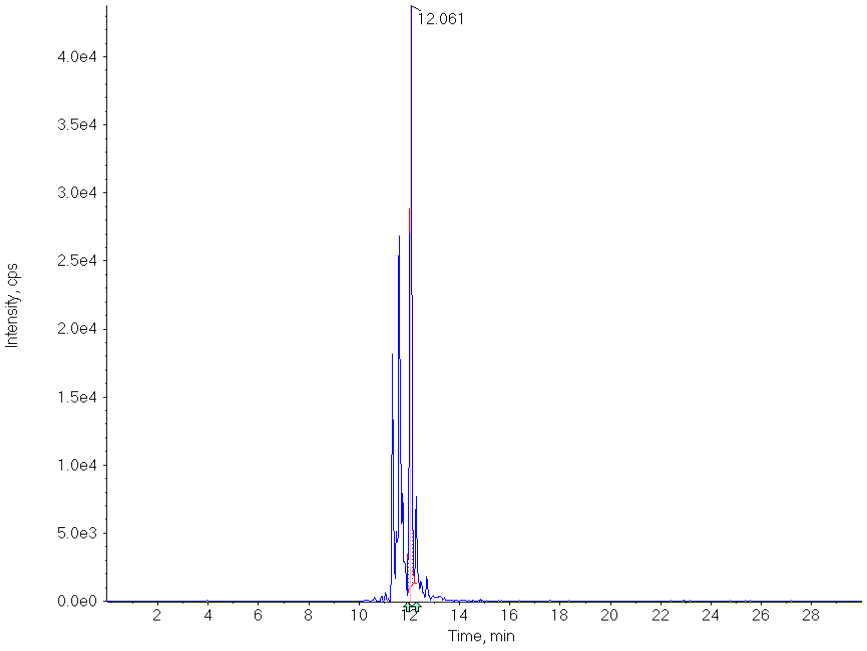

Acquired / Library MSMS

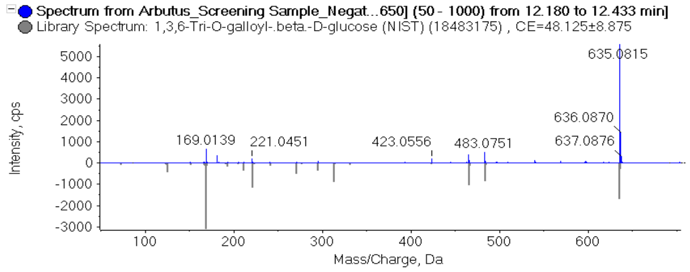

Acquired / Theoretical MS

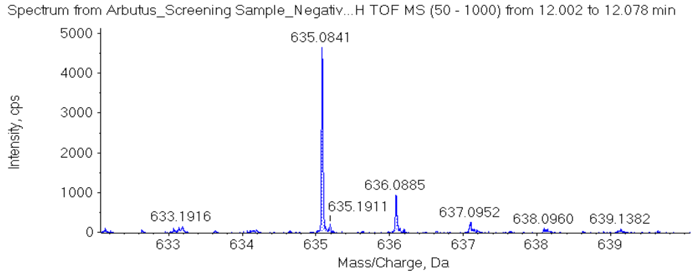

Cyanidin-3-*O*-glucoside

449.1037 / 12.12 [M-H2O-H]- (Mass/FragMass/RT/Isotope/Library/Formula/Ion Ratio)

Retention Time: 12.10 minutes
Precursor m/z : 449.1047
Fit (%) 79.9% **RFit (%)** 54.6%

Exp RT: 12.12 minutes
Analyte Name:
449.1037 / 12.12 [M-H2O-H]-

Collision Energy = 35 ± 15 eV

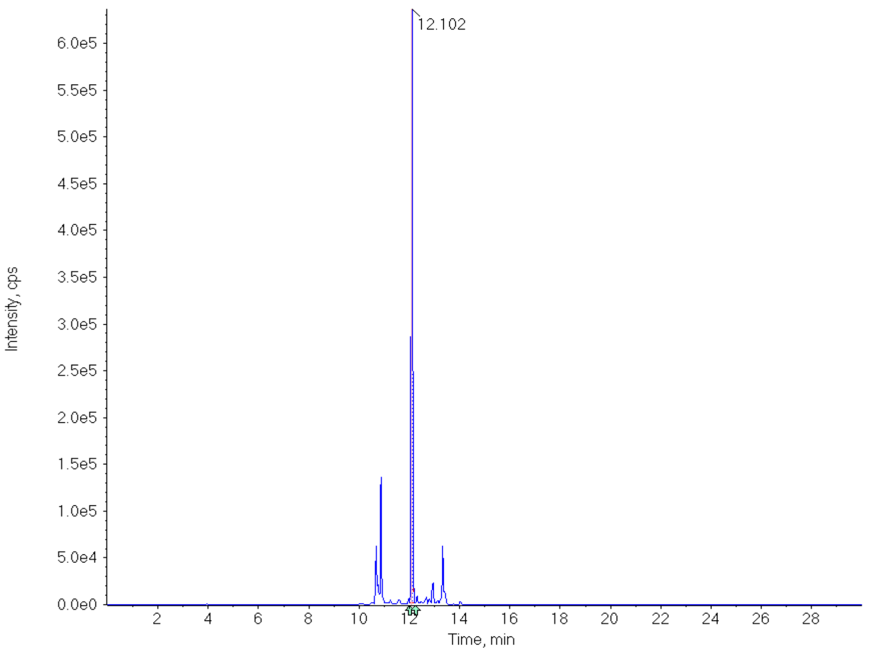

Acquired / Library MSMS

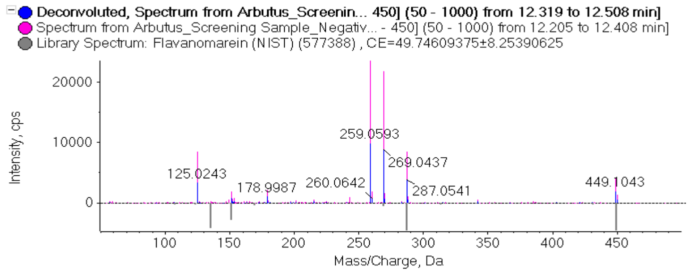

Acquired / Theoretical MS

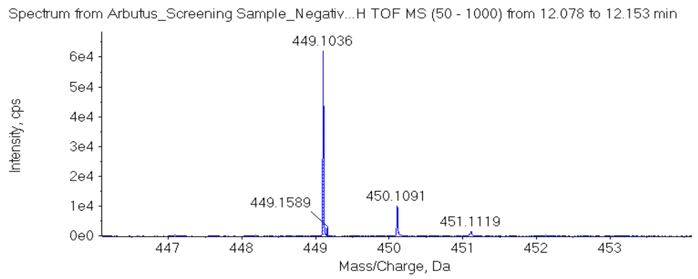

Methyl gallate

183.0285 / 12.23 [M+FA-H]-

(Mass/FragMass/RT/Isotope/Library/Formula/Ion Ratio)

Retention Time: 12.23 minutes
Precursor m/z : 183.0296
Fit (%) 100.0% **RFit (%)** 97.2%

Exp RT: 12.23 minutes
Analyte Name:
183.0285 / 12.23 [M+FA-H]-

Collision Energy = 35 ± 15 eV

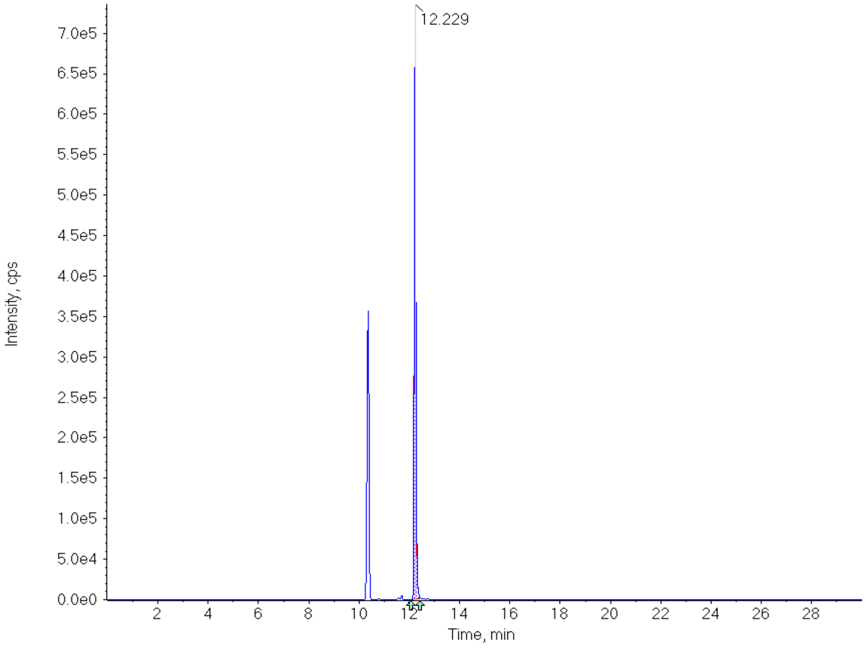

Acquired / Library MSMS

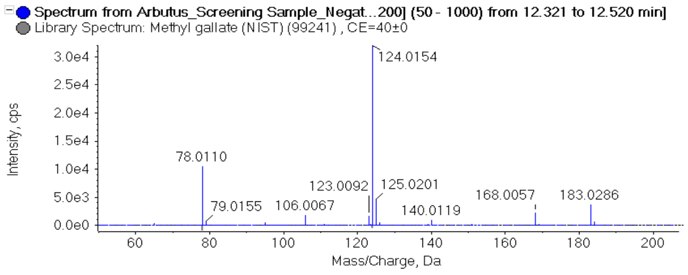

Acquired / Theoretical MS

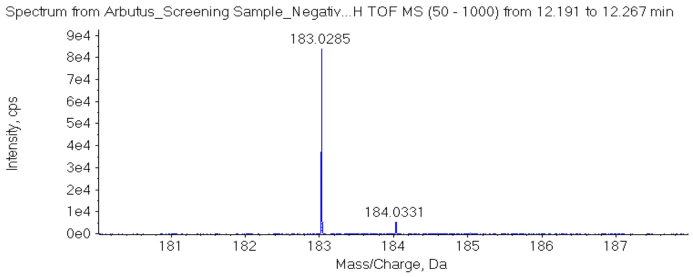

3-Hydroxy benzoic acid

137.0236 / 12.38 [M-H]- (Mass/FragMass/RT/Isotope/Library/Formula/Ion Ratio)

Retention Time: 12.40 minutes
Precursor m/z : 137.0247
Fit (%) 99.2% **RFit (%)** 98.6%

Exp RT: 12.38 minutes
Analyte Name:
137.0236 / 12.38 [M-H]-

Collision Energy = 35 ± 15 eV

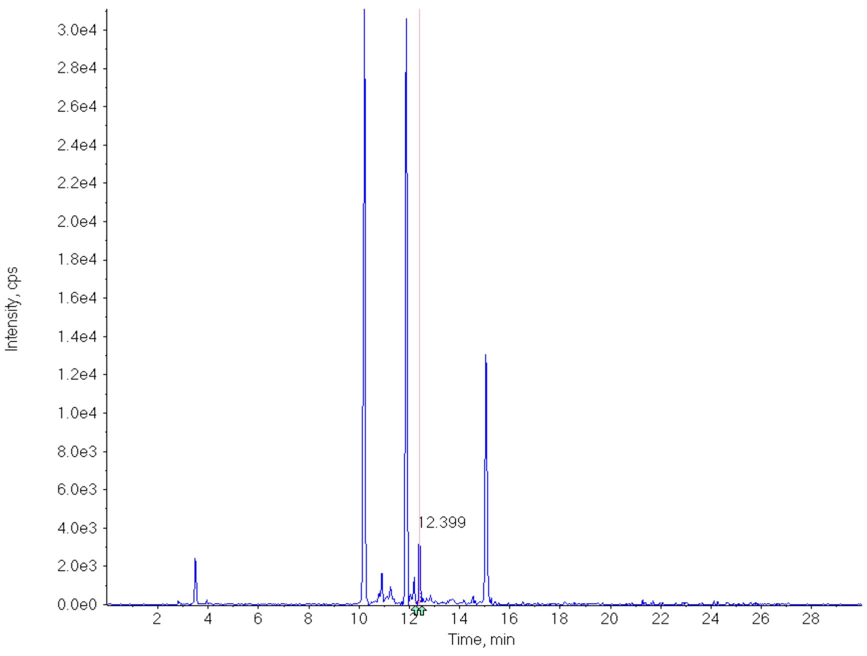

Acquired / Library MSMS

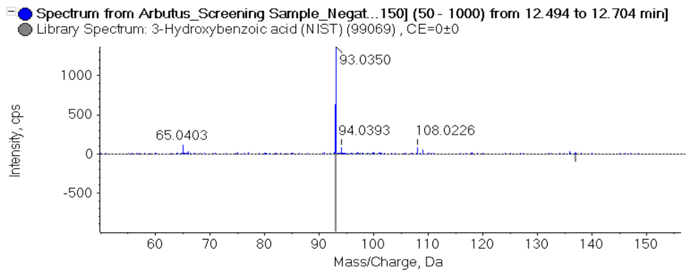

Acquired / Theoretical MS

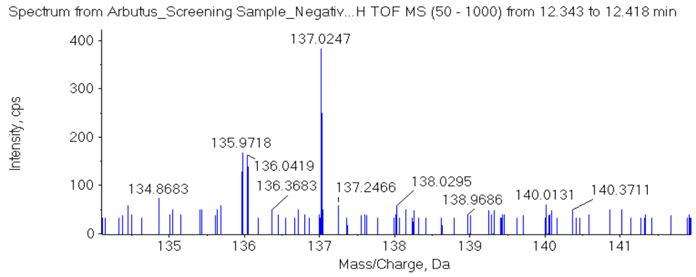

479.0793 / 12.38 (Mass/FragMass/RT/Isotope/Library/Formula/Ion Ratio)

Retention Time: 12.38 minutes
Precursor m/z : 479.0804
Fit (%) 99.0% **RFit (%)** 96.1%

Exp RT: 12.38 minutes
Analyte Name:
479.0793 / 12.38

Collision Energy = 35 ± 15 eV

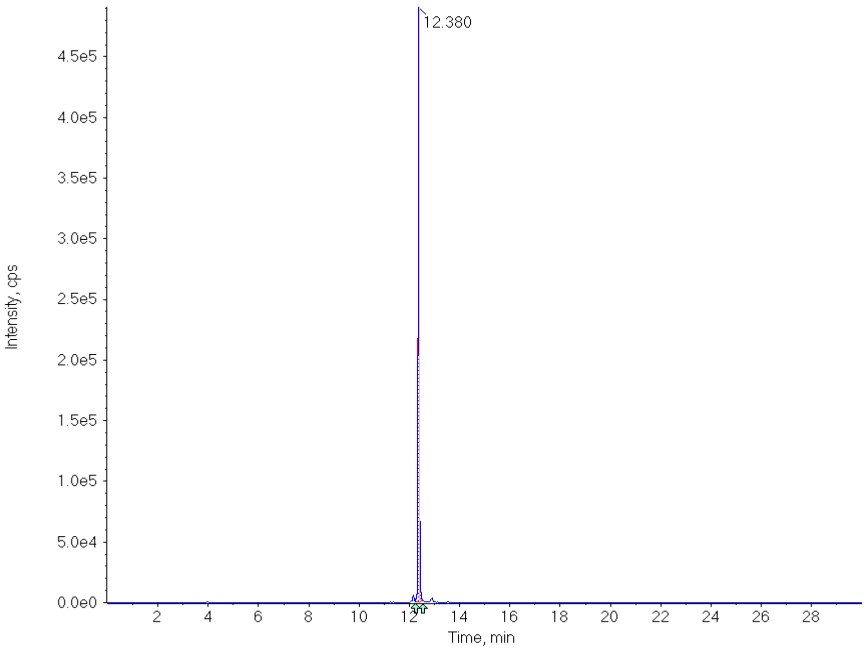

Acquired / Library MSMS

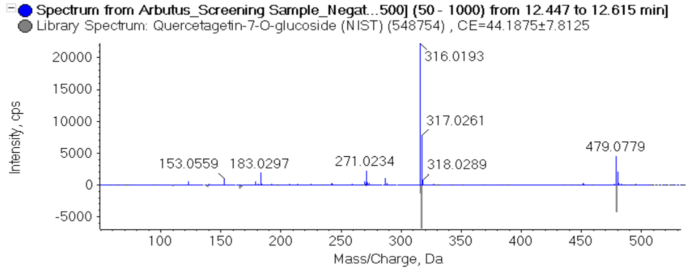

Acquired / Theoretical MS

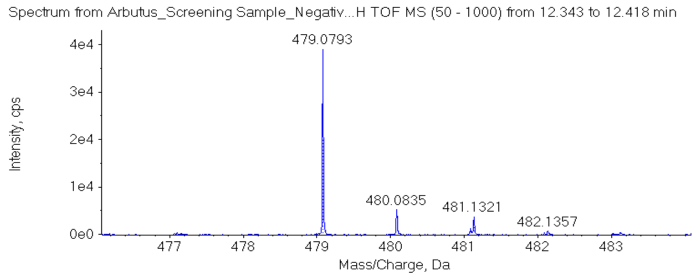

Quercetin 3-*O*- rutinoside

609.1396 / 12.49

(Mass/FragMass/RT/Isotope/Library/Formula/Ion Ratio)

**Retention Time:** 12.49 minutes	**Exp RT:** 12.49 minutes
**Precursor m/z :** 609.1407	**Analyte Name:**
**Fit (%)** 97.5% **RFit (%)** 94.4%	609.1396 / 12.49

Collision Energy = 35 ± 15 eV

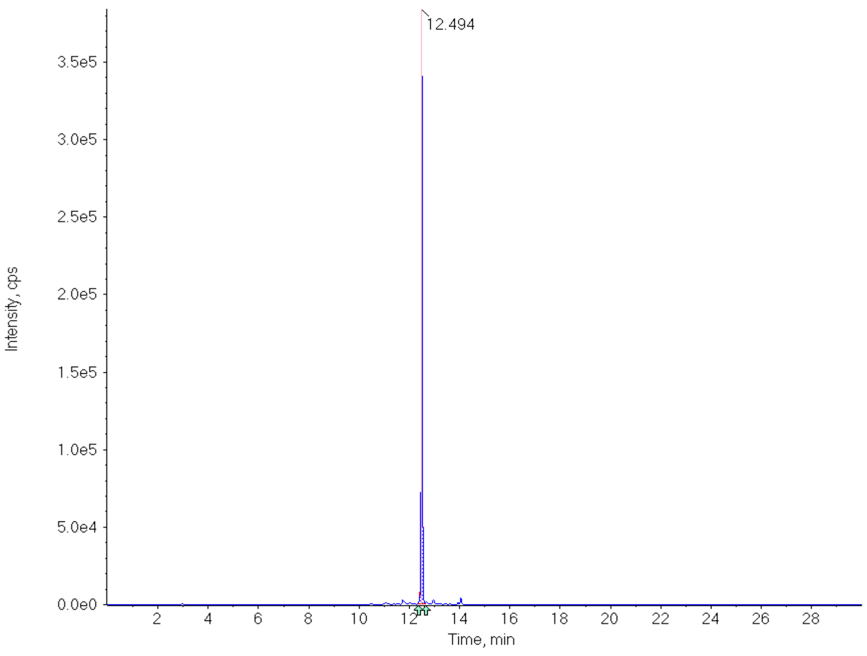

Acquired / Library MSMS

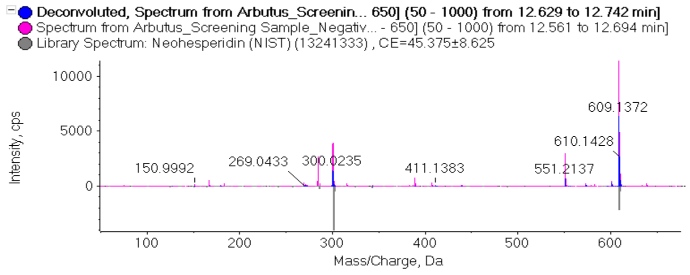

Acquired / Theoretical MS

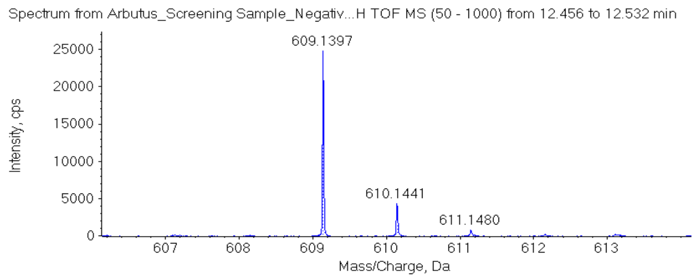

Quercetin-*O*-galloyl-glucoside

615.0919 / 12.61 (Mass/FragMass/RT/Isotope/Library/Formula/Ion Ratio)

Retention Time: 12.62 minutes
Precursor m/z : 615.0930
Fit (%) 97.2% **RFit (%)** 12.6%

Exp RT: 12.61 minutes
Analyte Name:
615.0919 / 12.61

Collision Energy = 35 ± 15 eV

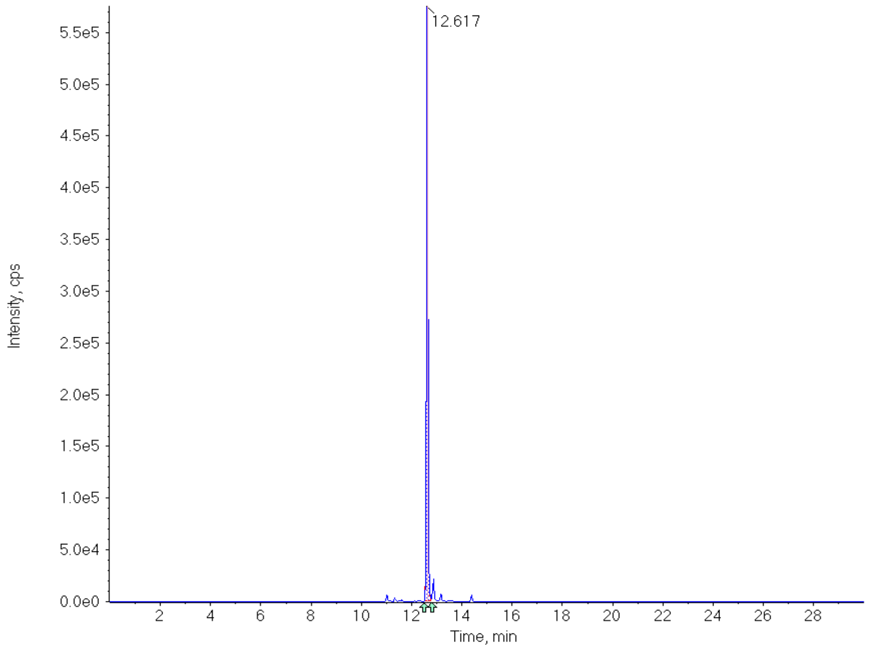

Acquired / Library MSMS

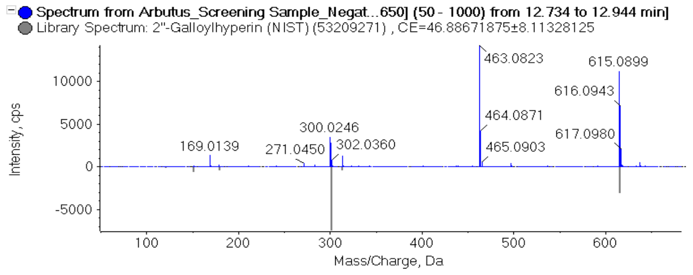

Acquired / Theoretical MS

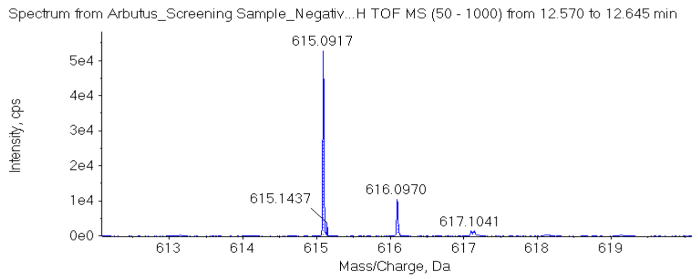

Luteolin-7-glucoside

447.0904 / 12.80 [M+FA-H]- (Mass/FragMass/RT/Isotope/Library/Formula/Ion Ratio)

Retention Time: 12.81 minutes
Precursor m/z : 447.0915
Fit (%) 100.0% **RFit (%)** 52.5%

Exp RT: 12.80 minutes
Analyte Name:
447.0904 / 12.80 [M+FA-H]-

Collision Energy = 35 ± 15 eV

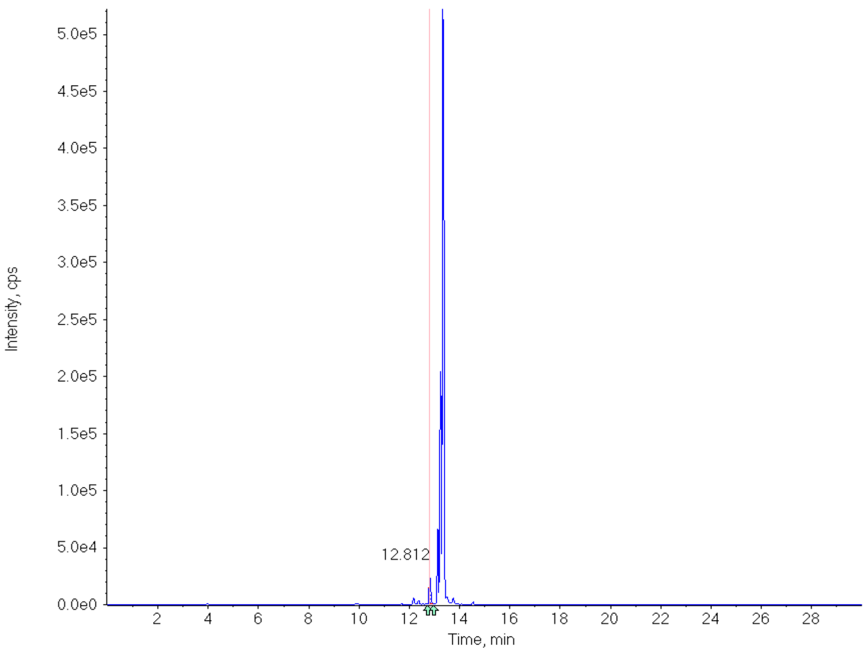

Acquired / Library MSMS

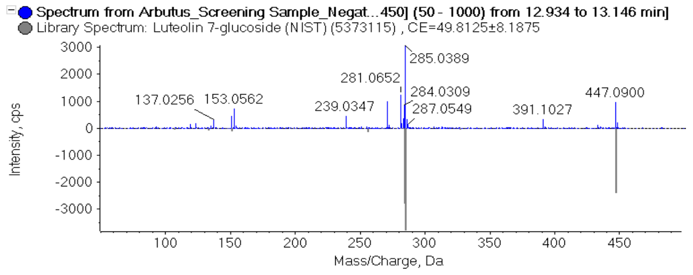

Acquired / Theoretical MS

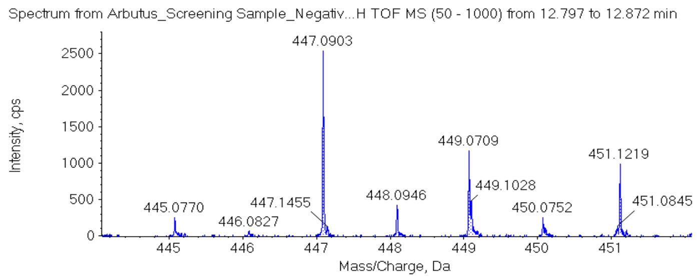

Quercetin-3-*O*-glucoside (Isoquercitin)

463.0822 / 12.83 (Mass/FragMass/RT/Isotope/Library/Formula/Ion Ratio)

**Retention Time:** 12.83 minutes	**Exp RT:** 12.83 minutes
**Precursor m/z :** 463.0833	**Analyte Name:**
**Fit (%)** 99.6% **RFit (%)** 90.9%	463.0822 / 12.83

Collision Energy = 35 ± 15 eV

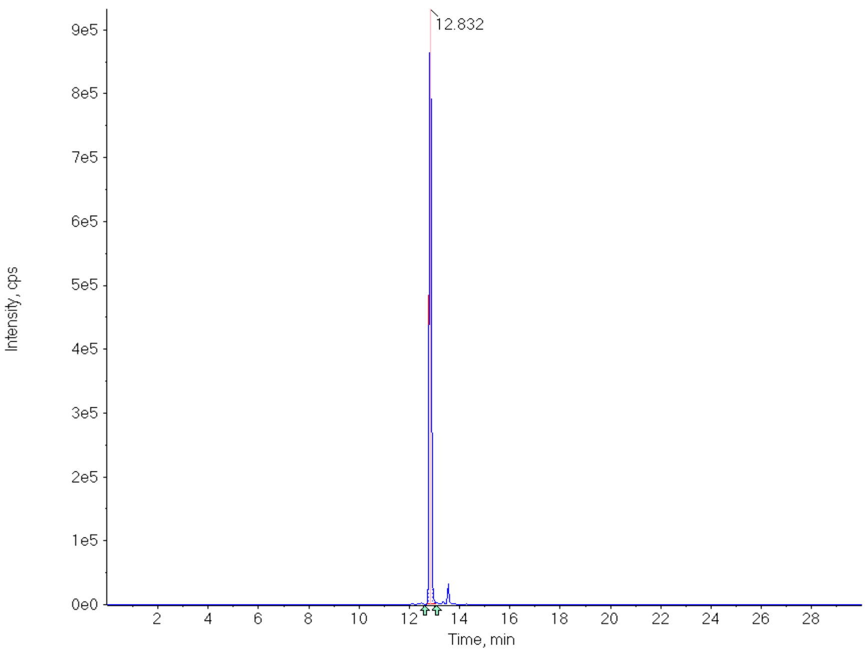

Acquired / Library MSMS

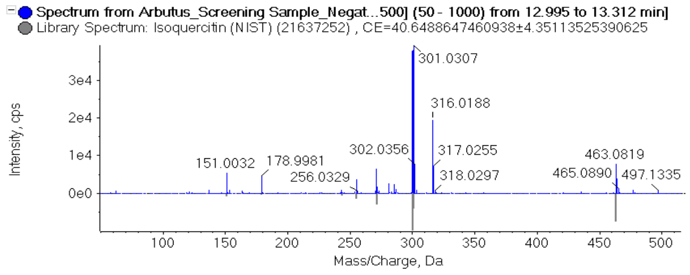

Acquired / Theoretical MS

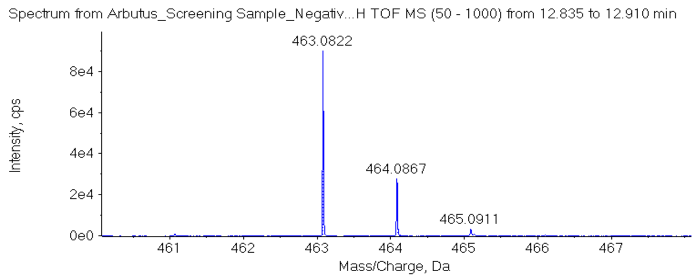

Ellagic acid

300.9972 / 13.06

(Mass/FragMass/RT/Isotope/Library/Formula/Ion Ratio)

Retention Time: 13.07 minutes
Precursor m/z : 300.9983
Fit (%) 96.5% **RFit (%)** 70.7%

Exp RT: 13.06 minutes
Analyte Name:
300.9972 / 13.06

Collision Energy = 35 ± 15 eV

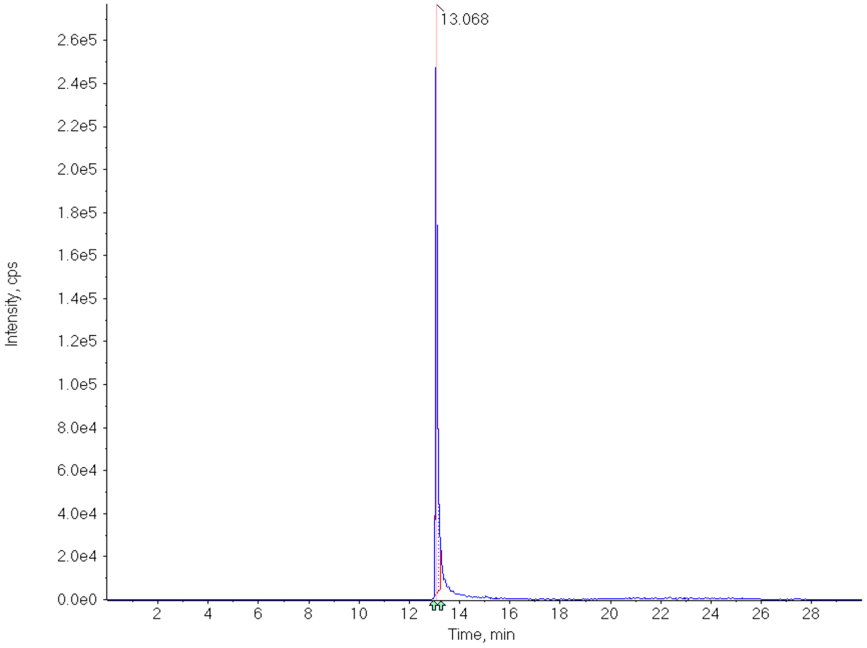

Acquired / Library MSMS

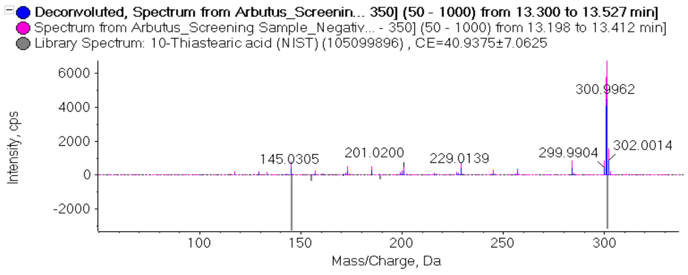

Acquired / Theoretical MS

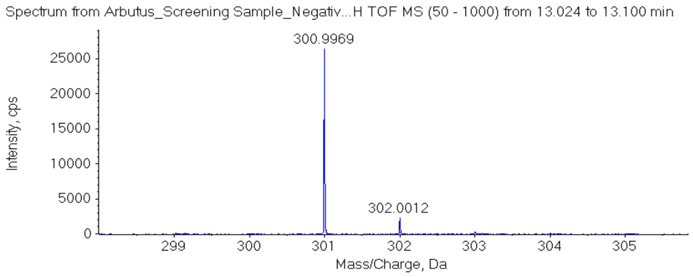

Quercetin-3-*O*-(arabinoside/xyloside)

.0743 / 13.21 (Mass/FragMass/RT/Isotope/Library/Formula/Ion Ratio)

Retention Time: 13.21 minutes
Precursor m/z : 433.0754
Fit (%) 80.1% **RFit (%)** 94.2%

Exp RT: 13.21 minutes
Analyte Name:
433.0743 / 13.21

Collision Energy = 35 ± 15 eV

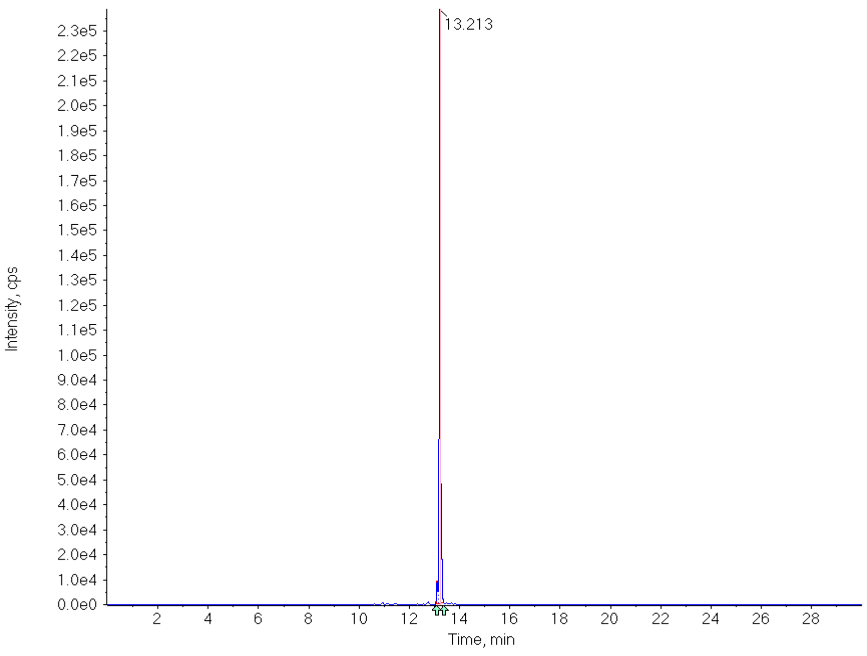

Acquired / Library MSMS

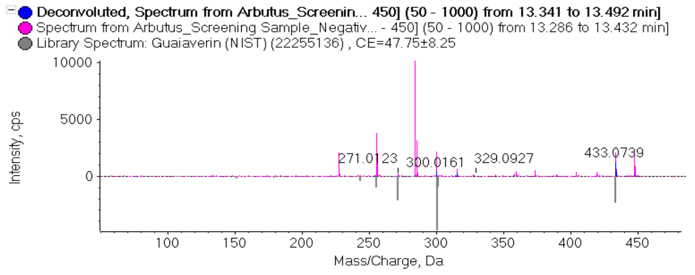

Acquired / Theoretical MS

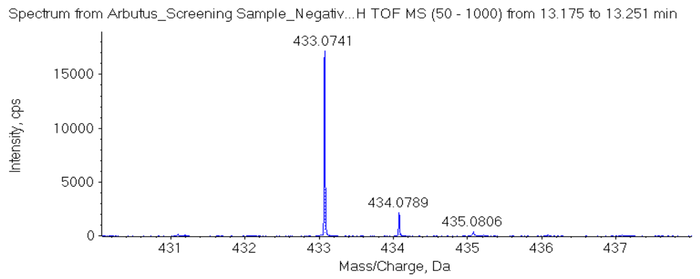

Isorhamnetin 3-*O*-glucoside

477.1003 / 13.29 (Mass/FragMass/RT/Isotope/Library/Formula/Ion Ratio)

Retention Time: 13.29 minutes
Precursor m/z : 477.1014
Fit (%) 42.7% **RFit (%)** 77.5%

Exp RT: 13.29 minutes
Analyte Name:
477.1003 / 13.29

Collision Energy = 35 ± 15 eV

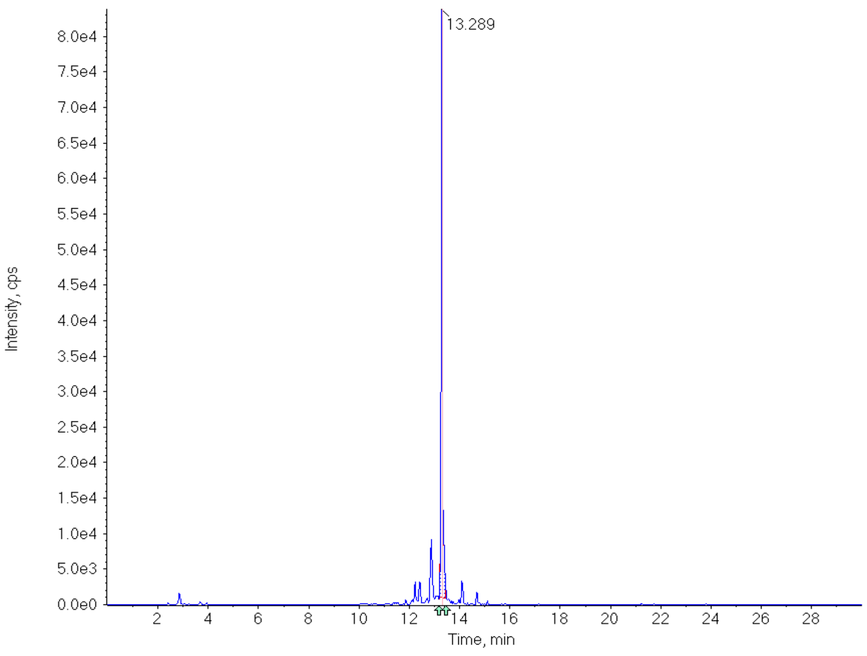

Acquired / Library MSMS

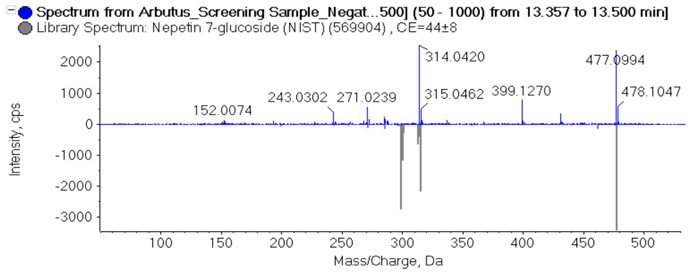

Acquired / Theoretical MS

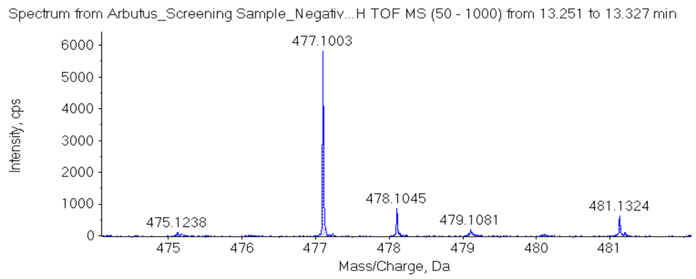

Quercetin 3-*O*-rhamnoside (Quercitrin)

447.0884 / 13.33 (Mass/FragMass/RT/Isotope/Library/Formula/Ion Ratio)

**Retention Time:** 13.34 minutes	**Exp RT:** 13.33 minutes
**Precursor m/z :** 447.0895	**Analyte Name:**
**Fit (%)** 100.0% **RFit (%)** 97.3%	447.0884 / 13.33

Collision Energy = 35 ± 15 eV

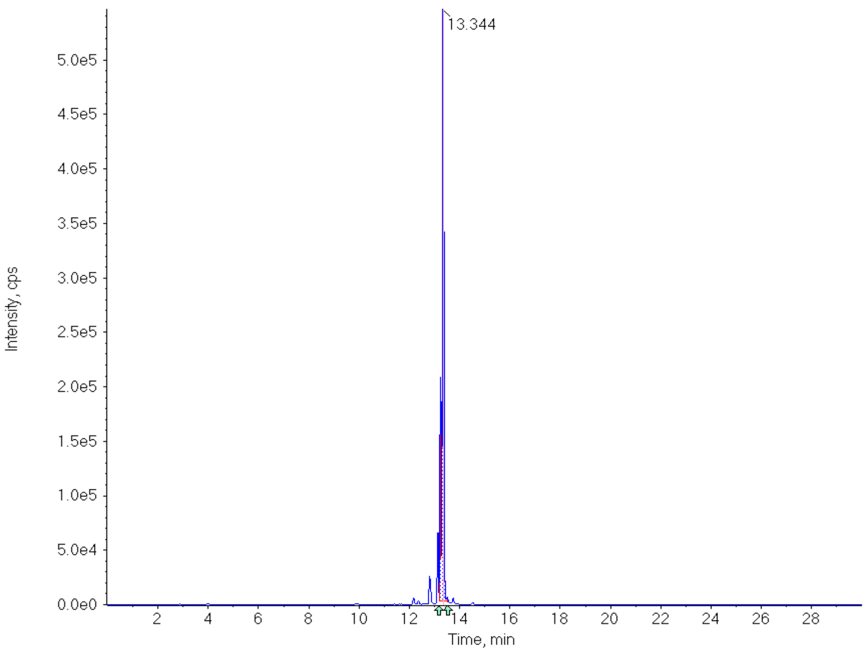

Acquired / Library MSMS

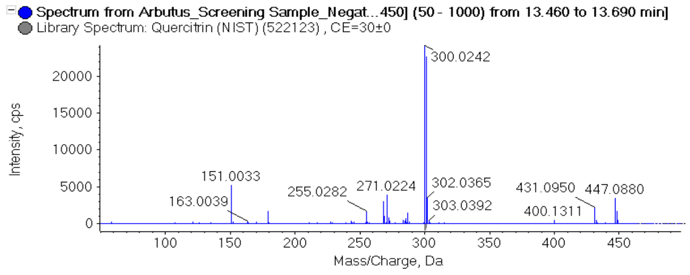

Acquired / Theoretical MS

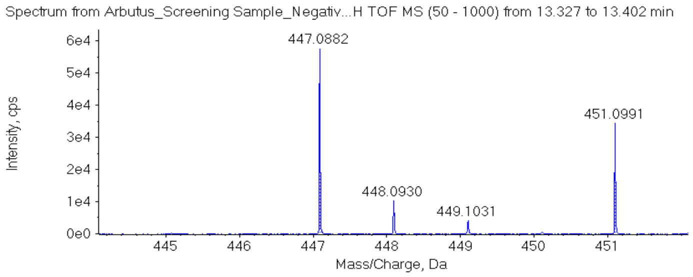

Myricetin rhamnoside

463.0851 / 13.55

(Mass/FragMass/RT/Isotope/Library/Formula/Ion Ratio)

Retention Time: 13.55 minutes
Precursor m/z : 463.0862
Fit (%) 99.0% **RFit (%)** 96.3%

Exp RT: 13.55 minutes
Analyte Name:
463.0851 / 13.55

Collision Energy = 35 ± 15 eV

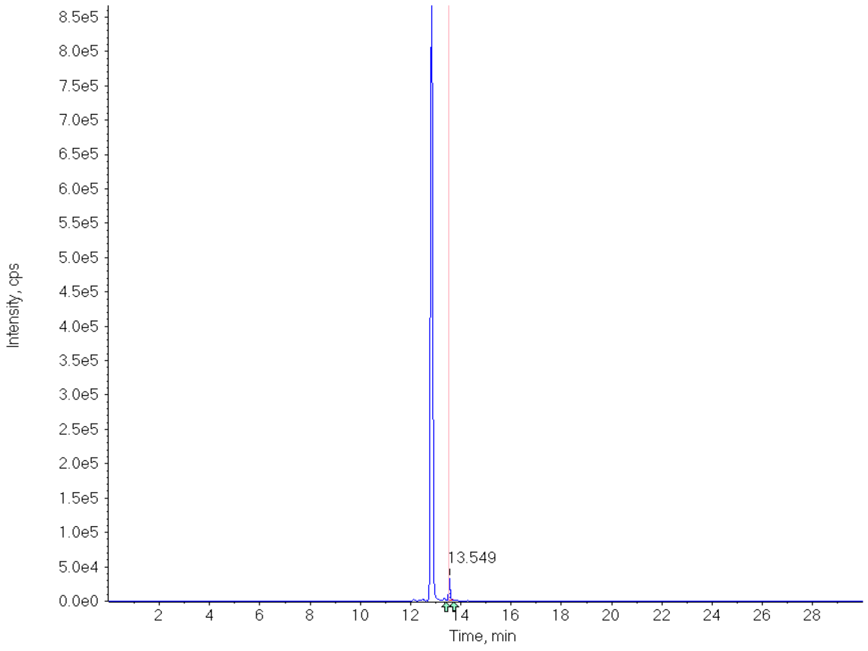

Acquired / Library MSMS

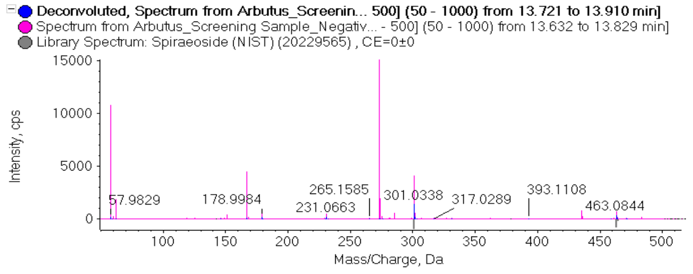

Acquired / Theoretical MS

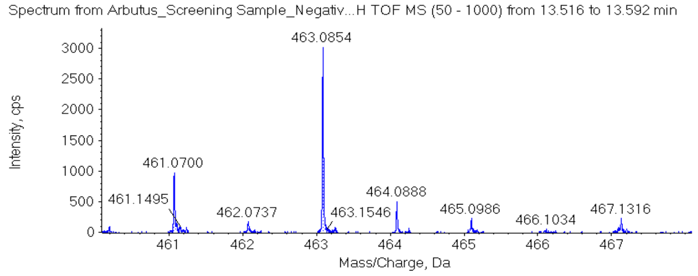

Vitexin

431.0963 / 13.82

(Mass/FragMass/RT/Isotope/Library/Formula/Ion Ratio)

**Retention Time:** 13.82 minutes	**Exp RT:** 13.82 minutes
**Precursor m/z :** 431.0974	**Analyte Name:**
**Fit (%)** N/A **RFit (%)** N/A	431.0963 / 13.82

Collision Energy = 35 ± 15 eV

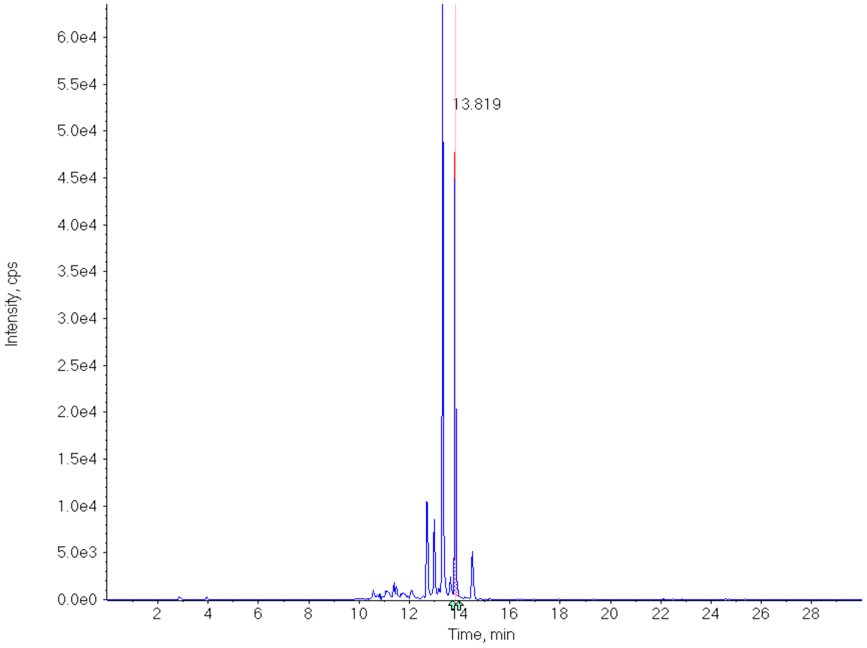

Acquired / Library MSMS

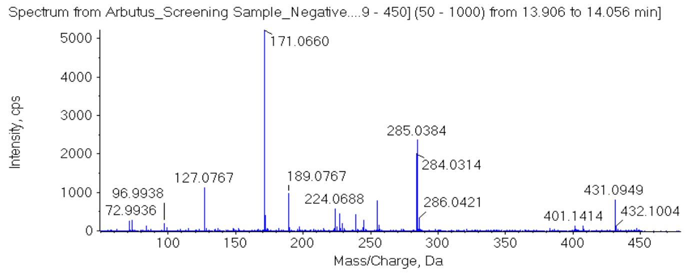

Acquired / Theoretical MS

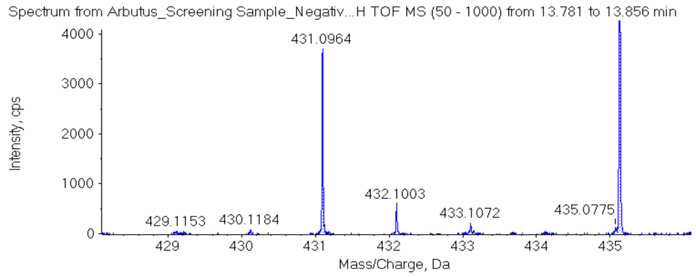

Myricetin

317.0288 / 14.08

(Mass/FragMass/RT/Isotope/Library/Formula/Ion Ratio)

**Retention Time:** 14.08 minutes	**Exp RT:** 14.08 minutes
**Precursor m/z :** 317.0299	**Analyte Name:**
**Fit (%)** 94.0% **RFit (%)** 98.0%	317.0288 / 14.08

Collision Energy = 35 ± 15 eV

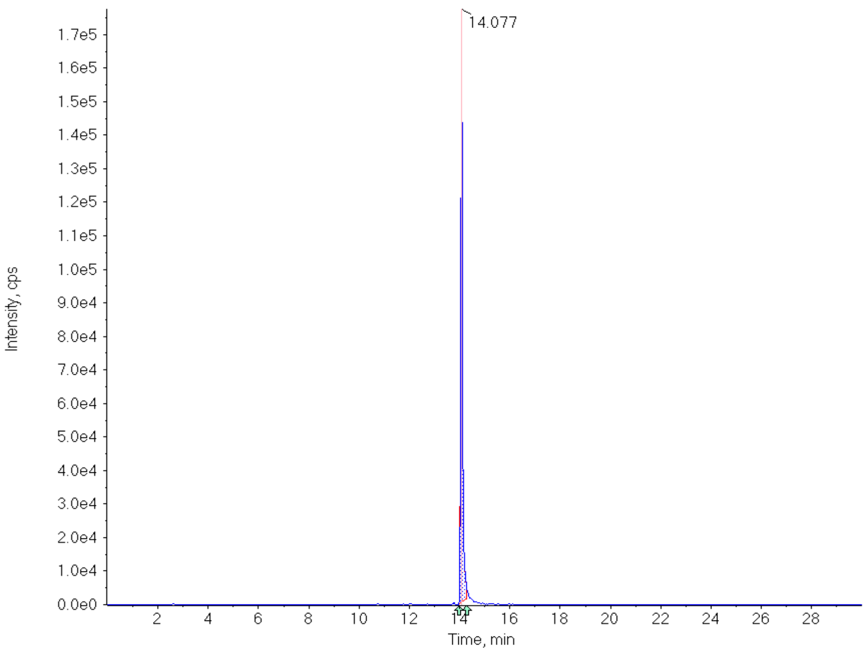

Acquired / Library MSMS

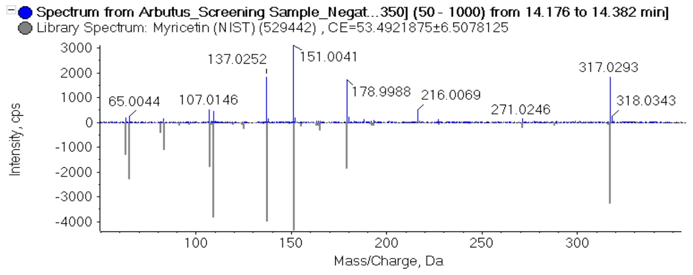

Acquired / Theoretical MS

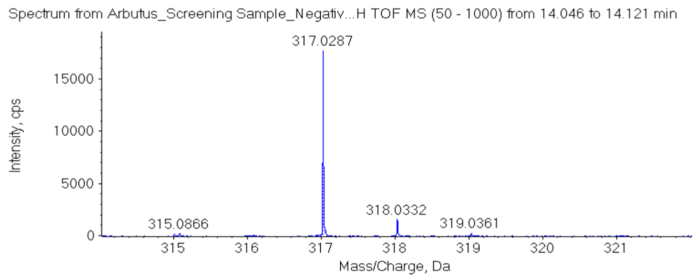

Catechin

289.0817 / 14.12 [M+AcO-H]- (Mass/FragMass/RT/Isotope/Library/Formula/Ion Ratio)

Retention Time: 14.41 minutes
Precursor m/z : 289.0828
Fit (%) N/A **RFit (%)** N/A

Exp RT: 14.12 minutes
Analyte Name:
289.0817 / 14.12 [M+AcO-H]-

Collision Energy = 35 ± 15 eV

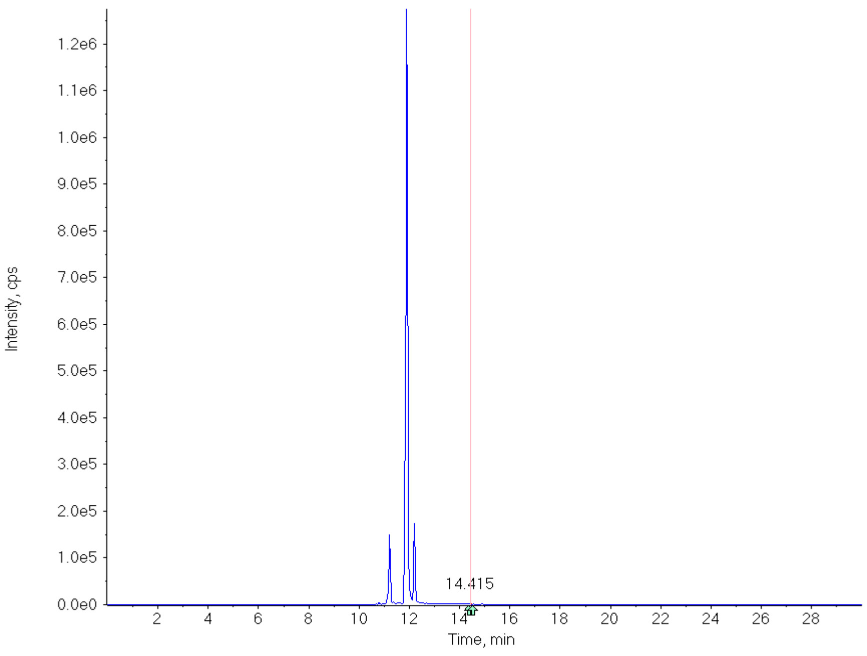

Acquired / Library MSMS

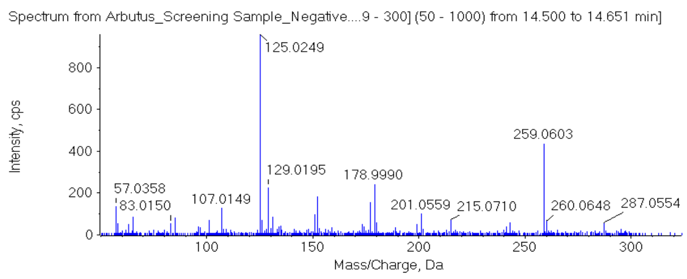

Acquired / Theoretical MS

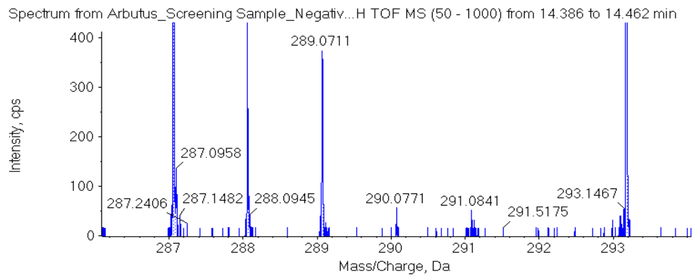

Quercetin

301.0344 / 15.03 (Mass/FragMass/RT/Isotope/Library/Formula/Ion Ratio)

Retention Time: 15.04 minutes
Precursor m/z : 301.0355
Fit (%) 91.1% **RFit (%)** 98.0%

Exp RT: 15.03 minutes
Analyte Name:
301.0344 / 15.03

Collision Energy = 35 ± 15 eV

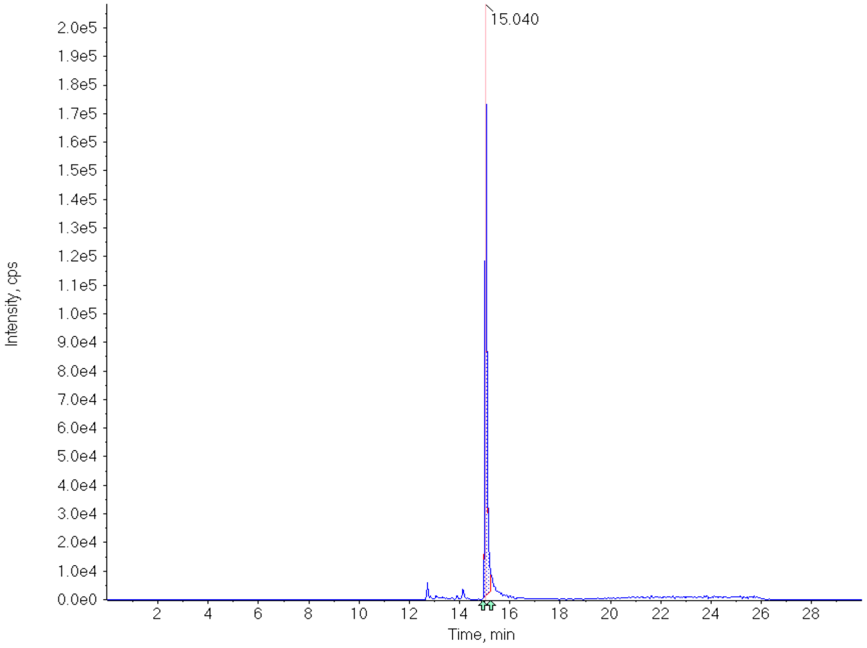

Acquired / Library MSMS

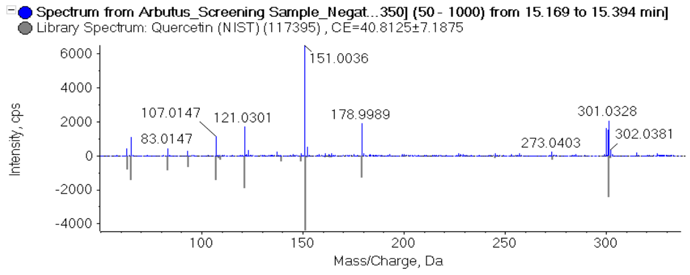

Acquired / Theoretical MS

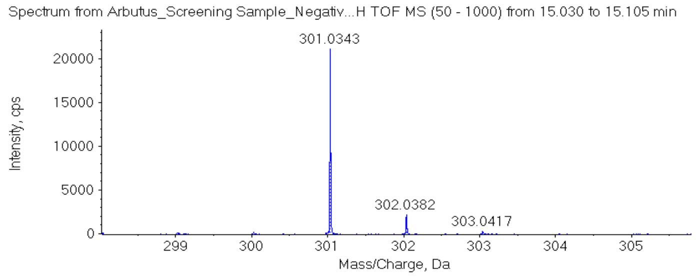

Luteolin

285.0420 / 15.22 (Mass/FragMass/RT/Isotope/Library/Formula/Ion Ratio)

**Retention Time:** 14.99 minutes	**Exp RT:** 15.22 minutes
**Precursor m/z :** 285.0431	**Analyte Name:**
**Fit (%)** 95.3% **RFit (%)** 92.5%	285.0420 / 15.22

Collision Energy = 35 ± 15 eV

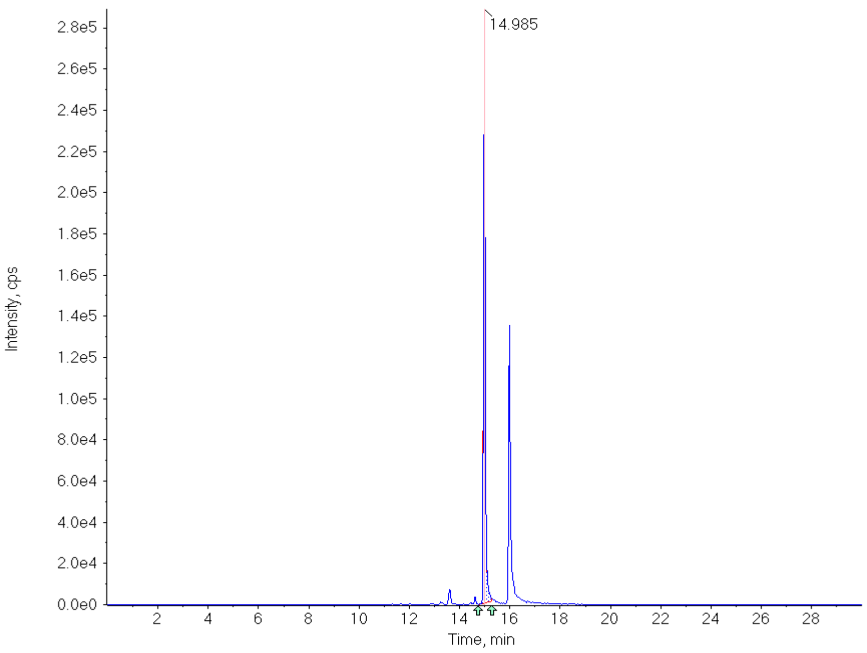

Acquired / Library MSMS

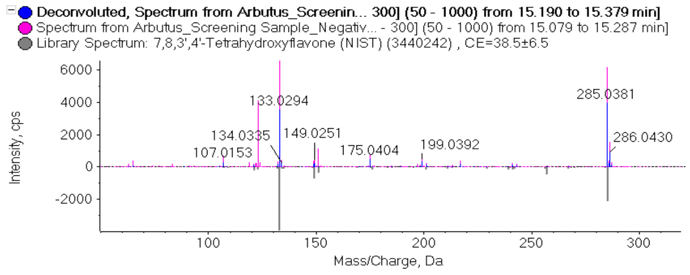

Acquired / Theoretical MS

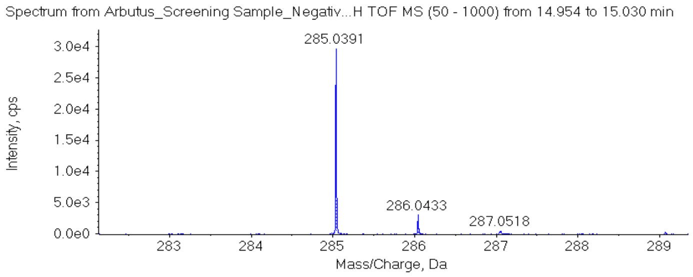

Kaempferol

285.0396 / 15.98 (Mass/FragMass/RT/Isotope/Library/Formula/Ion Ratio)

Retention Time: 15.98 minutes
Precursor m/z : 285.0407
Fit (%) 99.5% **RFit (%)** 95.0%

Exp RT: 15.98 minutes
Analyte Name:
285.0396 / 15.98

Collision Energy = 35 ± 15 eV

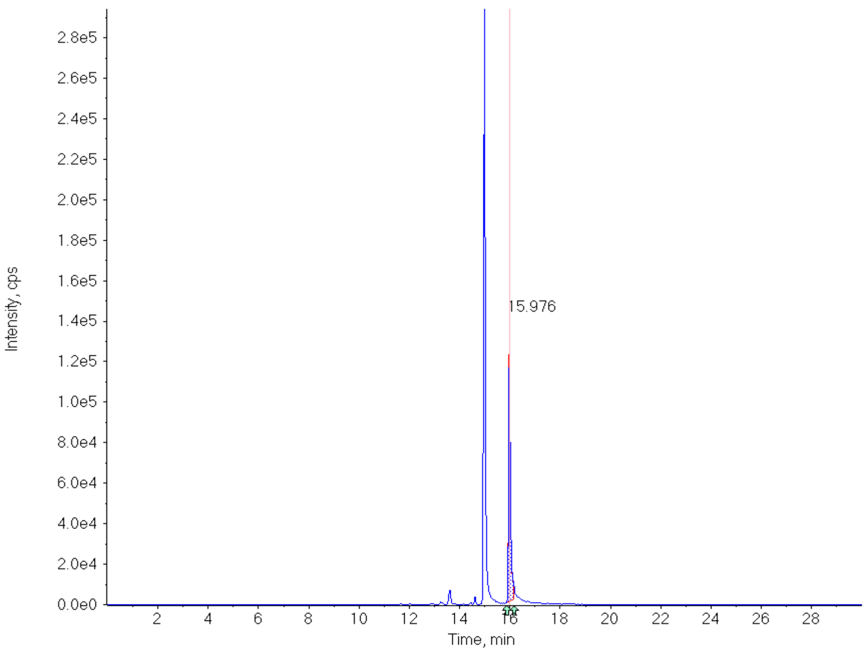

Acquired / Library MSMS

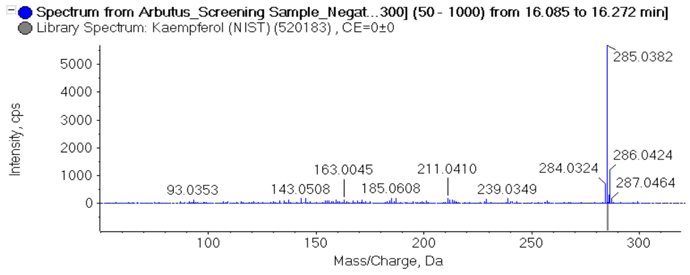

Acquired / Theoretical MS

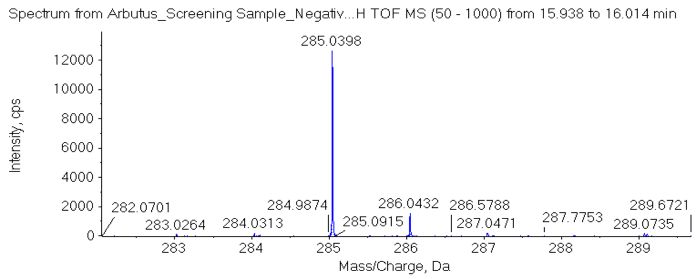

Supplement: Supplemental Material [file IENZ_A_2293639_SM6360.pdf]
